# RNP2 of RNA Recognition Motif 1 Plays a Central Role in the Aberrant Modification of TDP-43

**DOI:** 10.1371/journal.pone.0066966

**Published:** 2013-06-28

**Authors:** Shinnosuke Takagi, Yohei Iguchi, Masahisa Katsuno, Shinsuke Ishigaki, Kensuke Ikenaka, Yusuke Fujioka, Daiyu Honda, Jun-ichi Niwa, Fumiaki Tanaka, Hirohisa Watanabe, Hiroaki Adachi, Gen Sobue

**Affiliations:** 1 Department of Neurology, Nagoya University Graduate School of Medicine, Nagoya, Japan; 2 Stroke Center, Aichi Medical University, Aichi, Japan; 3 Department of Neurology and Stroke Medicine, Yokohama City University Graduate School of Medicine, Yokohama, Japan; International Centre for Genetic Engineering and Biotechnology, Italy

## Abstract

Phosphorylated and truncated TAR DNA-binding protein-43 (TDP-43) is a major component of ubiquitinated cytoplasmic inclusions in neuronal and glial cells of two TDP-43 proteinopathies, amyotrophic lateral sclerosis and frontotemporal lobar degeneration. Modifications of TDP-43 are thus considered to play an important role in the pathogenesis of TDP-43 proteinopathies. However, both the initial cause of these abnormal modifications and the TDP-43 region responsible for its aggregation remain uncertain. Here we report that the 32 kDa C-terminal fragment of TDP-43, which lacks the RNP2 motif of RNA binding motif 1 (RRM1), formed aggregates in cultured cells, and that similar phenotypes were obtained when the RNP2 motif was either deleted from or mutated in full-length TDP-43. These aggregations were ubiquitinated, phosphorylated and truncated, and sequestered the 25 kDa C-terminal TDP-43 fragment seen in the neurons of TDP-43 proteinopathy patients. In addition, incubation with RNase decreased the solubility of TDP-43 in cell lysates. These findings suggest that the RNP2 motif of RRM1 plays a substantial role in pathological TDP-43 modifications and that it is possible that disruption of RNA binding may underlie the process of TDP-43 aggregation.

## Introduction

Amyotrophic lateral sclerosis (ALS) and certain forms of frontotemporal lobar degeneration (FTLD) with ubiquitin-positive but tau-negative inclusions have been considered to be a single disease spectrum of TAR DNA-binding protein 43 (TDP-43) proteinopathy since 2006, when this protein was reported to be a major component of ubiquitin-positive inclusions in the affected neuronal and glial cells of both ALS and FTLD [1]–[3]. The identification of missense mutations of *TARDBP*, the gene encoding TDP-43, in familial and sporadic ALS and/or FTLD patients further confirmed the importance of this molecule in the pathogenesis of TDP-43 proteinopathies [4]–[7].

Although TDP-43 normally localizes to the nucleus, it is distributed from nucleus to cytoplasm or neurite and forms aggregates that mainly consist of C-terminal fragments (CTFs) in the affected neurons of TDP-43 proteinopathy patients. In addition, aberrantly aggregated TDP-43 is hyperphosphorylated at multiple C-terminal sites [8]. The fact that most TDP-43 proteinopathy cases are sporadic suggests that exogenous factors induce the post-translational modifications of TDP-43 that are seen in the disease.

Although it does not fully recapitulate the pathological features of TDP-43 proteinopathies, artificial axonal damage induces transient cytoplasmic distribution of TDP-43 in mouse motor neurons [9], [10], and several stress conditions, including oxidative stress and suppression of the ubiquitin-proteasome system, cause aberrant modifications of TDP-43 in cultured cell lines or primary neurons [11]–[15]. In addition, a motor neuron-specific disruption of proteasomes results in the cytosolic distribution of TDP-43 in a mouse model [16]. Finally, the repeat expansion of GGGGCC in C9orf72, as well as mutations in UBQLN2, VCP, PGRN, or OPTN, lead to neurodegeneration with TDP-43-positive neuronal inclusions [17]–[22].

These findings provide us with a clue for elucidating the mechanism of these modifications. Nevertheless, both the initial cause of these abnormal modifications and the region of TDP-43 responsible for its aggregation remain unknown. Here we report that RNP2 in RNA binding motif 1 (RRM1) plays a substantial role in the pathological TDP-43 modifications that are seen in TDP-43 proteinopathies.

## Materials and Methods

### Cell Culture and Treatment

Mouse NSC34 motor neuron-like cells (a kind gift of N.R. Cashman, University of British Columbia, Vancouver, Canada) [23] and human embryonic kidney 293 (HEK293) cells were cultured in a humidified atmosphere of 95% air/5% CO2 in a 37°C incubator in Dulbecco's Modified Eagle's Medium (DMEM) supplemented with 10% fetal bovine serum (FBS). In both NSC34 and HEK293 cells, the transfections of the intended plasmids were performed using Lipofectamine 2000 (Invitrogen), according to the manufacturer's instructions. Before performing subsequent experiments, the cells were incubated for 48 h after transfection.

### DNA Constructs

Human wild-type TDP-43 (WT-TDP-43) (accession number NM 007375) cDNA was amplified by PCR from human spinal cord cDNA as previously described [11]. The PCR product was cloned into the pENTR/D-TOPO vector (Invitrogen). For the TDP-43 truncated fragments, amplified PCR products (for primers see Table S1) from the WT-TDP-43 vector were cloned into the pENTR/D-TOPO vector. For delta RNP2 TDP-43 (ΔRNP2), mutated RNP2 TDP-43 (mtRNP2), mutated RNP1 TDP-43 (mRNP1), delta RRM1 TDP-43 (ΔRRM1), and delta nuclear localization signal (NLS) TDP-43 (dNLS) vectors, primers containing mutant substitutions (Table S2) were used to mutagenize WT-TDP-43 (KOD-Plus-Mutagenesis kit; Toyobo). The entry vectors of each mutant TDP-43 vector were transferred into either a pcDNA6.2/N-EmGFP-DEST or pcDNA3.1/nV5-DEST vector using the Gateway LR Clonase II enzyme mix (Invitrogen). The sequences of all constructs were verified using the CEQ 8000 genetic analysis system (Beckman Coulter). The collection of autopsied human tissue and its use for this study were approved by the Ethics Committee of Nagoya University Graduate School of Medicine, and written informed consent was obtained from the patients’ next-of-kin. The experimental procedure involving the human subject was conducted in conformance with the principles expressed in the Declaration of Helsinki.

### Immunoblot Analysis

We used NE-PER Nuclear Cytoplasmic Reagents (Thermo Fisher Scientific) for the analysis of the cytoplasmic/nuclear ratio. For analysis of protein solubility, cells cultured in 10-cm plates were lysed in 1 ml RIPA buffer (Thermo Fisher Scientific). Lysates were sonicated and centrifuged at 100,000 *g* for 15 min. To prevent carryover, the pellets were washed with RIPA buffer, followed by sonication and centrifugation. RIPA-insoluble pellets were lysed in 100 µl urea buffer (7 M urea, 2 M thiourea, 4% CHAPS, 30 mM Tris, pH 8.5), sonicated, and centrifuged at 100,000 *g* for 15 min.

After the denaturation, 5 µl of each sample was separated by SDS-PAGE (5%–20% gradient gel) and the proteins were then transferred to Hybond-P membranes (Amersham Pharmacia Biotech). The membranes were blocked with 5% skimmed milk in Tris-buffered saline containing 0.05% Tween-20 and incubated with the intended primary antibodies. The primary antibodies used were as follows: anti-TDP-43 rabbit polyclonal antibody (1∶1000; ProteinTech); anti-pTDP-43 (phospho Ser409/410) rabbit polyclonal antibody (1∶1000; Cosmo Bio); anti-ubiquitin mouse monoclonal antibody (MBL); anti-histone H1 mouse monoclonal antibody (1∶500; Millipore); anti-GAPDH mouse monoclonal antibody (1∶2000; Temecula); anti-GFP mouse monoclonal antibody (1∶2000; MBL); and anti-V5 mouse monoclonal antibody (1∶5000; Invitrogen). For the anti-ubiquitin antibody, the membranes were fixed with 0.05% glutaraldehyde/0.1M KH_2_PO_4_ and blocked with 4% BSA. Secondary antibody probing and detection were performed using ECL Plus detection reagents (GE Healthcare). Chemiluminescence signals were digitized (LAS-3000 Imaging System; Fujifilm) and band intensities were quantified using Multi Gauge software (version 3.0; Fujifilm).

### Immunocytochemistry

NSC34 cells were fixed in 4% paraformaldehyde, incubated in PBS containing 0.3% Triton X-100 for 5 min, blocked with Image-iT FX signal enhancer (Invitrogen), and incubated overnight at 4°C with anti-TDP-43 rabbit polyclonal antibody (1∶1000; ProteinTech), anti-pTDP-43 (phospho Ser409/410) rabbit polyclonal antibody (1∶500; Cosmo Bio), anti-TIAR mouse monoclonal antibody (1∶500; BD Transduction Laboratories, Milan, Italy), anti-ubiquitin mouse monoclonal antibody (1∶100; MBL), anti-V5 rabbit polyclonal antibody (1∶1000; Bethyl) or anti-V5 mouse monoclonal antibody (Invitrogen). After washing, samples were incubated with Alexa-488-conjugated goat anti-mouse IgG and Alexa-555-conjugated goat anti-rabbit IgG (both at 1∶1000; Invitrogen) for 60 min, mounted with Prolong Gold antifade reagent with DAPI (Invitrogen), and then imaged with a confocal microscope (LSM710; Zeiss).

For the counting of inclusion-bearing cells, we randomly selected 100 transfected cells from three separate experiments. The colocalization coefficient, which reflects the fraction of green pixels that are also positive for red pixels, was calculated using the Zeiss LSM software. We calculated the colocalization coefficient by randomly selected 10 fields from three separate experiments. To obtain images for calculating the colocalization coefficient, the settings of the confocal microscopy and the threshold of positive/negative fluorescence was fixed within each experiment.

### Immunoprecipitation

Transfected HEK293 cells were washed with PBS and lysed in immunoprecipitation buffer (Thermo Fisher Scientific). After sonication on ice, the samples were agitated for 30 min at 4°C. The samples were centrifuged and supernatants were incubated with magnetic beads: anti-V5 magnetic beads (MBL), anti-GFP magnetic beads (MBL), and anti-ubiquitin magnetic beads (MBL). Samples were rotated overnight at 4°C. Immunoprecipitates were separated by SDS-PAGE (5%–20% gradient gel). Western blotting was performed using anti-V5-HRP antibody (MBL) and anti-GFP-HRP antibody (MBL).

### Ribonucleoprotein Immunoprecipitation

Ribonucleoprotein (RNP) immunoprecipitation was performed using a RIP assay kit (MBL), according to the manufacturer's instructions. RNA concentrations were measured with a Nanodrop (Thermo Fisher Scientific). Electrophoresis of precipitated RNA was performed with a Bioanalyzer (Agilent Technologies) according to the manufacturer's instructions. For analysis of neurofilament light chain (hNFL) mRNA 3′UTR content, RNA obtained from immunoprecipitates was reverse transcribed into first-strand cDNA using SuperScript II reverse transcriptase (Invitrogen) and a PCR was performed with the following primers: ACCAACCAGTTGAGTTCCAGAT (forward) and GAATGATTCACATTGCCGTAGA (reverse).

### Effect of RNase on TDP-43 Solubility

For analysis of protein solubility with or without RNase, HEK293 cells cultured in 10-cm plates were lysed in 1 ml of Tris-saline (TS) buffer (50 mM Tris-HCl buffer, pH 7.5, 0.15 M NaCl, 5 mM EDTA, protein phosphatase inhibitors, and a protease inhibitor cocktail). Lysates were sonicated and then divided into two samples. RNase A (10 µg/ml) was added to one of the samples. Samples incubated for 0 and 15 h at 4°C were centrifuged at 100,000 *g* for 15 min. To prevent carryover, the pellets were washed with TS buffer, followed by sonication and centrifugation. TS-insoluble pellets were lysed in 1 ml of Triton X-100 (TX) buffer (TS buffer containing 1% Triton X-100), sonicated, and centrifuged at 100,000 *g* for 15 min. The pellets were washed with TX buffer, followed by sonication and centrifugation. TX-insoluble pellets were lysed in 500 µl of Sarkosyl (Sar) buffer (TS buffer containing 1% Sarkosyl), sonicated and centrifuged at 100,000 *g* for 15 min. Sar-insoluble pellets were lysed in 100 µl of urea buffer.

### Statistical Analyses

Statistical analyses were performed using GraphPad Prism software (GraphPad software inc.). Biochemical data were statistically analyzed using a Student's *t*-test or one-factor factorial ANOVA followed by Tukey post hoc tests. A *p* value of 0.05 or less was considered to be statistically significant.

## Results

### Intracellular Localization of CTFs of TDP-43 in NSC34 Cells

**Figure 1:**
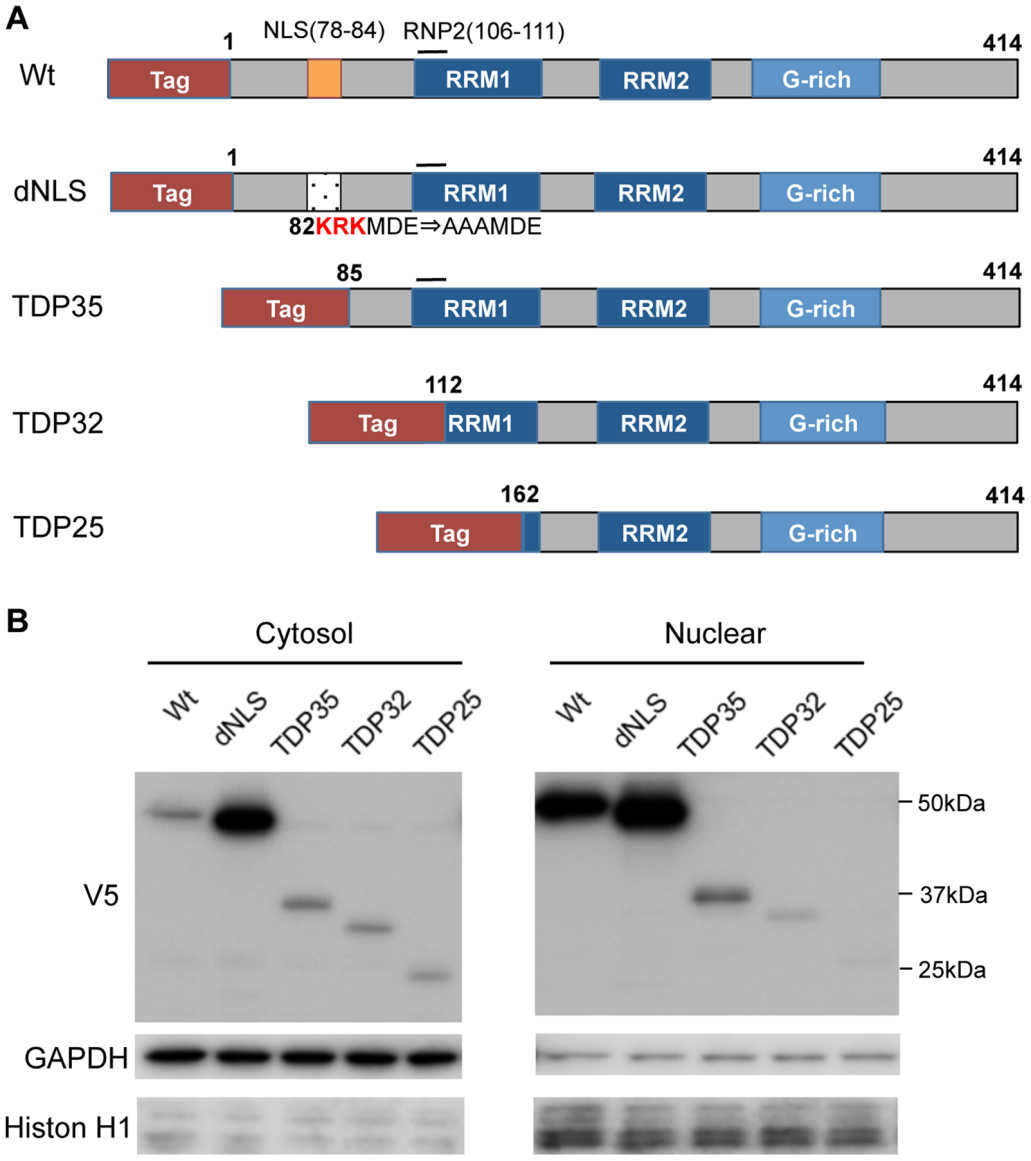
Intracellular localizations of TDP-43 lacking the NLS and of CTFs of TDP-43. (A) Structures of wild-type (Wt), NLS-disrupted mutant (dNLS) and CTFs (TDP35, TDP32 and TDP25). (B) Immunoblots of the cytosol and nuclear fractions from HEK293 cells expressing Wt, dNLS, TDP35, TDP32, or TDP25.

To identify the region of TDP-43 that is responsible for the pathological modification of this protein, we created various TDP-43 mutants and investigated their intracellular localizations. In particular, given that the CTFs of TDP-43 form aggregates in the cytoplasm of affected neurons, we focused on the mutant TDP-43 in which the NLS is disrupted (dNLS) and on TDP-43 CTFs: 35 kDa (TDP35), 32 kDa (TDP32), and 25 kDa (TDP25) fragments (Fig. 1A). We examined TDP32, which does not contain RNP2 motif (aa 106–111), to access the effect of RNA-binding on the pathological modification, since RNP2 motif is indispensable for RNA-binding of RRM1 [24]. Each form of TDP-43 was transfected into NSC34 cells for immunocytochemical analysis and into HEK293 cells for the analysis of their localization using fractionated immunoblots.

Although wild-type TDP-43 showed a nuclear-dominant distribution, the dNLS mutant localized to the cytosol more preferentially than wild-type TDP-43 (Fig. 1B). The CTFs of TDP-43, all of which lack the NLS, also showed a cytosolic localization. In particular, the short CTFs, TDP32 and TDP25, had a strong propensity to distribute in the cytosol. We also found that the total soluble protein levels of TDP32 and TDP25 were less than that of TDP35, suggesting that these two CTFs may be insoluble or rapidly degraded.

### Ubiquitination of TDP-43 CTFs

**Figure 2:**
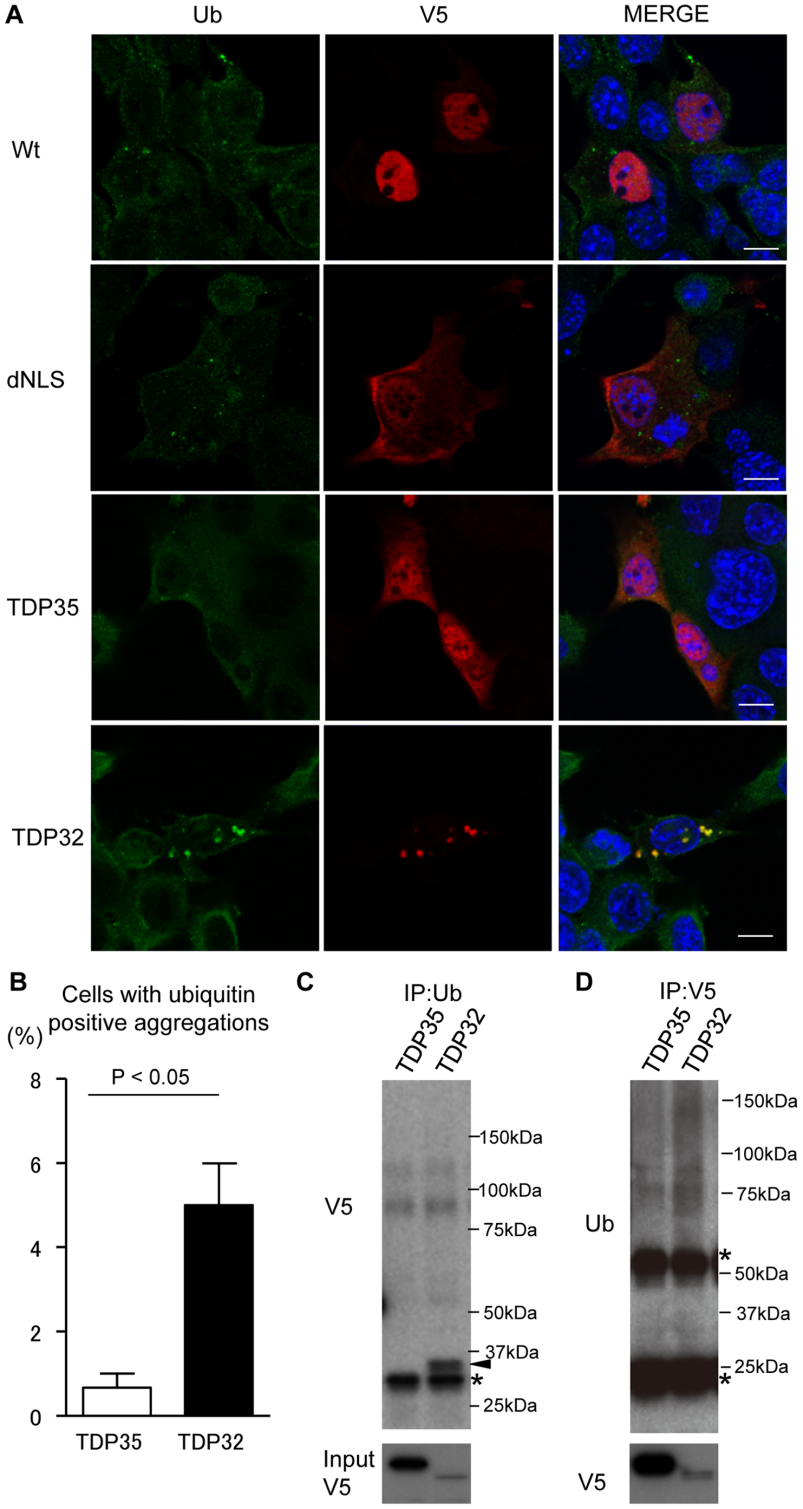
Ubiquitination of TDP-43 CTFs. (A) Immunocytochemistry of NSC34 cells expressing Wt, dNLS, TDP35, or TDP32. Cells were stained with anti-ubiquitin antibody (green), anti-V5 antibody (red), and DAPI (blue). Scale bar = 5 µm. (B) Percentage of cells with ubiquitin-positive aggregates. Error bars indicate SEM (n = 3). The percentage of TDP32-expressing cells containing ubiquitin-positive aggregates was significantly higher than that of cells expressing TDP35 (*p*<0.05). (C) Immunoprecipitations with anti-ubiquitin antibody. The immunoreactivity of V5 was only detected in the TDP32 lane (arrow head). Asterisk indicates non-specific signal. (D) Immunoprecipitations with anti-V5 antibody. The ubiquitin-positive smear band was increased in the lane of TDP32 compared with that of TDP35. Asterisks indicate a heavy or light chain of IgG.

The relatively low steady-state levels of TDP32 and TDP25 prompted us to explore the possibility that these CTFs form aggregates. Given that the expression levels of TDP25 are substantially lower than other forms of TDP-43, we first focused on dNLS, TDP35, and TDP32. In immunocytochemical analysis using anti-ubiquitin and -V5 antibodies, wild-type TDP-43 chiefly localized to the nucleus, but a substantial amount of dNLS and TDP35 distributed to the cytosol (Fig. 2A). TDP32 also showed a cytosolic localization but formed aggregates that were stained with anti-ubiquitin antibody (Fig. 2A, B).

In contrast, ubiquitin-positive aggregates were virtually undetectable in the cells expressing TDP35 (Fig. 2B). Since TDP-43 is reported to form RNA-containing structures like stress granules, we examined the relationship between TDP-43 CTFs and T-cell-restricted intracellular antigen 1-related (TIAR) protein, a marker of stress granules. Although TDP35 occasionally colocalized with TIAR, the inclusions of TDP32 were distinct from anti-TIAR-stained RNA granules (Fig. S1).

To quantitatively analyze the relationship between ubiquitin and TDP-43 CTFs, we calculated the colocalization coefficient of ubiquitin and V5 immunofluorescence. We found that the colocalization coefficient of ubiquitin and V5 was significantly higher in TDP32-expressing cells than in those expressing TDP35 (*p*<0.001; Fig. S2). We also confirmed using immunoprecipitation the differential ubiquitination of TDP35 and TDP32. Anti-ubiquitin immunoprecipitates were only detected in the cells expressing TDP32, although the steady-state levels of TDP32 were far lower than those of TDP35 (Fig. 2C). Immunoblotting of anti-V5 immunoprecipitates also showed that the ubiquitin-positive smear was denser in the cells bearing TDP32 than in those expressing TDP35 (Fig. 2D). Together, these results suggest that TDP32, but not TDP35, forms ubiquitin-positive aggregates.

### Insolubilization and Phosphorylation of the 32 kDa CTF of TDP-43

**Figure 3:**
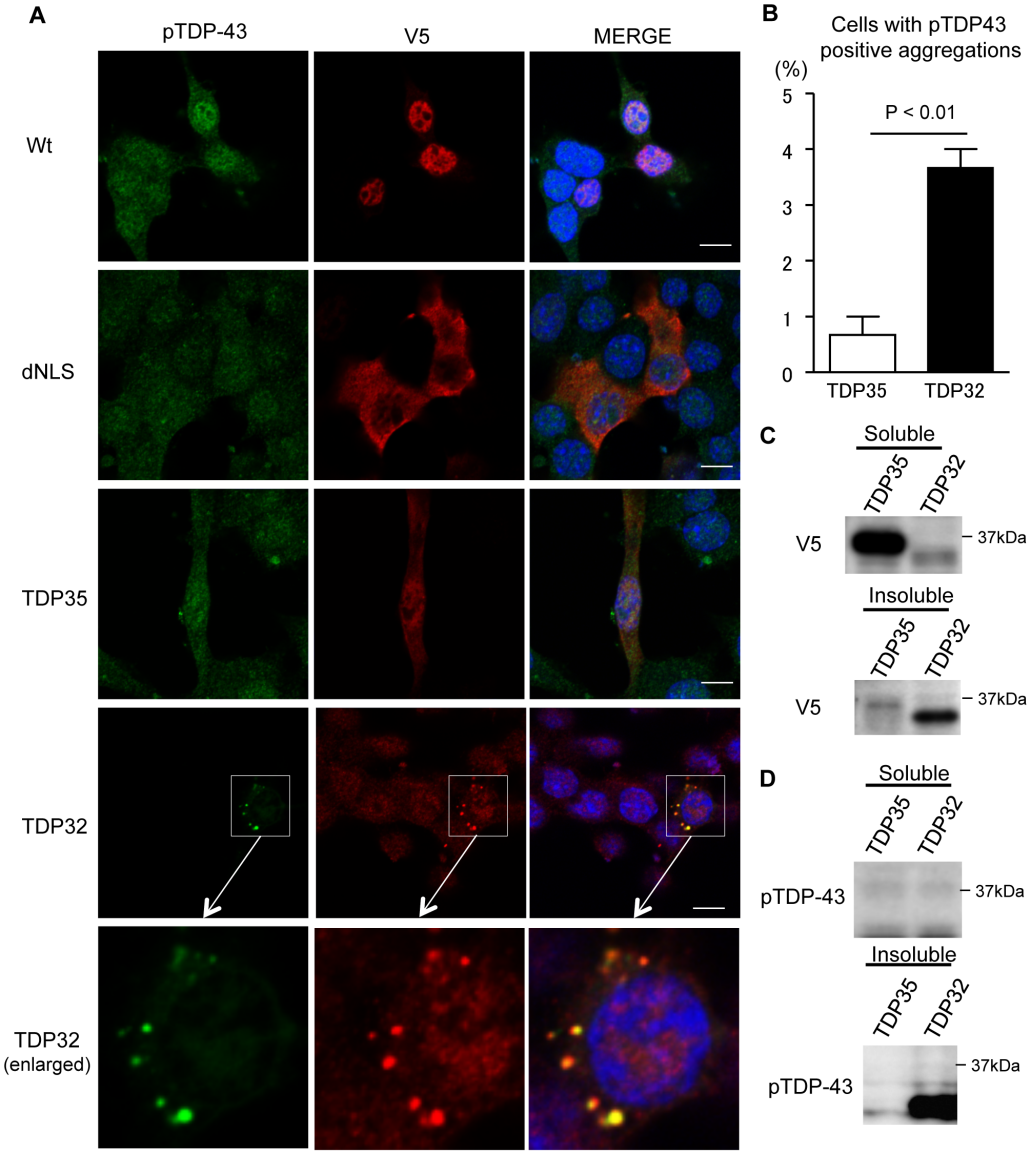
Phosphorylation and insolubilization of TDP32. (A) Immunocytochemistry of NSC34 cells expressing Wt, dNLS, TDP35, or TDP32. Cells were stained with anti-pTDP-43 antibody (green), anti-V5 antibody (red), and DAPI (blue). Scale bar = 5 µm. (B) Percentage of cells with pTDP-43-positive aggregates. Error bars indicate SEM (n = 3). The percentage of TDP32-expressing cells containing pTDP-43-positive aggregates was significantly higher than that of TDP35 (*p*<0.01). (C and D) Immunoblots of RIPA-soluble and -insoluble fractions from HEK293 cells expressing TDP35 and TDP32. The amount of insoluble TDP32 was higher than that of TDP35 (C). TDP32 in the RIPA-insoluble fraction was detected with anti-pTDP-43 antibody (D).

Like ubiquitination, insolubilization and phosphorylation are characteristics of TDP-43 proteinopathies. Therefore, we investigated TDP-43 CTF solubility and phosphorylation, finding that although the cytosolic aggregates of TDP32 were well stained with anti-phosphorylated TDP-43 (pTDP-43) antibody, the other forms showed no detectable pTDP-43-containing aggregates (Fig. 3A). The quantitative analysis also confirmed that the number of pTDP-43-positive aggregates was significantly higher in the cells expressing TDP32 than in those transfected with the TDP35 vector (*p*<0.01; Fig. 3B). The colocalization coefficient of TDP-43 CTFs and phosphorylated TDP-43 was also significantly higher in the cells expressing TDP32 than in those with TDP35 (*p*<0.001; Fig. S3). In the immunoblots performed using RIPA lysis buffer, the amount of insoluble TDP32 was higher than that of TDP35, whereas most of the TDP35 was solubilized by this buffer (Fig. 3C). TDP32 in the RIPA-insoluble fraction was also detected with an anti-pTDP-43 antibody (Fig. 3D).

### Disruption of the RNP2 Motif in TDP-43 Leads to Ubiquitin-positive Aggregate Formation

The phenotype of TDP32 was distinctly different from that of TDP35, although both of which lack the NLS, span amino acids 85–414 and 112–414, respectively (Fig. 1A). Although these two CTFs, and share a common structure with regard to the RRM2 and glycine-rich domains. The critical difference between these CTFs is the RNP2 (aa 106–111), the RNA binding motif at its N-terminal portion, which was included in TDP35 but not in TDP32. The contrast between TDP35 and TDP32 regarding modifications suggests that RNP2 motif is responsible for the induction of critical changes such as ubiquitination, phosphorylation, and insolubilization.

**Figure 4:**
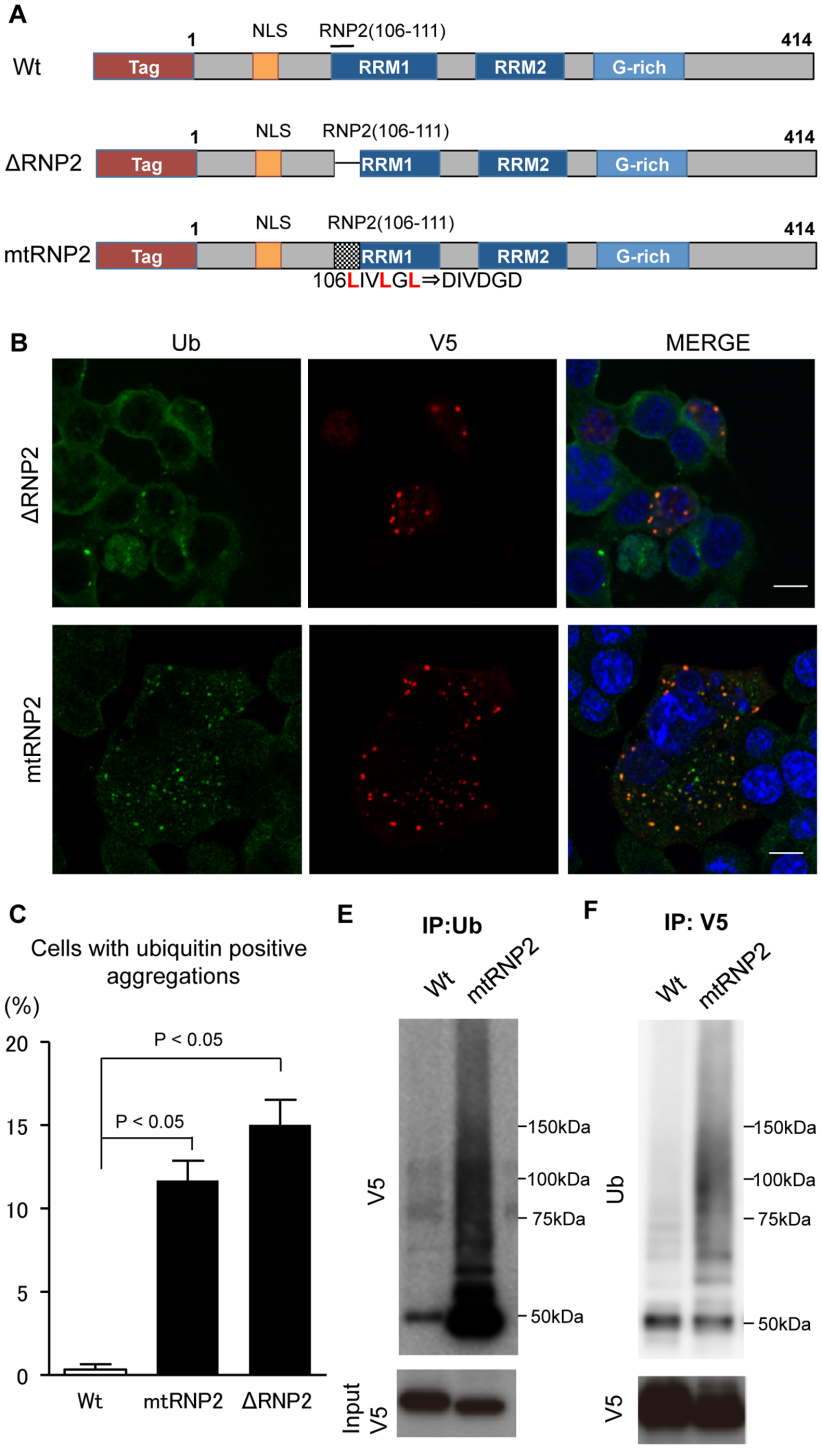
Disruption of the RNP2 motif leads to ubiquitin-positive aggregates of TDP-43. (A) Structures of Wt, ΔRNP2, and mtRNP2. (B) Immunocytochemistry of NSC34 cells expressing Wt, ΔRNP2, or mtRNP2. Cells were stained with anti-ubiquitin antibody (green), anti-V5 antibody (red), and DAPI (blue). Scale bar = 5 µm. (C) Percentage of cells with ubiquitin-positive aggregates. Error bars indicate SEM (n = 3). The percentage of mtRNP2 and ΔRNP2-expressing cells containing ubiquitin-positive aggregates was significantly higher than that of Wt-expressing cells (*p*<0.01 and *p*<0.001, respectively). (D) Immunoprecipitations with anti-ubiquitin antibody. The V5-positive smear band was evident in the mtRNP2 lane. (E) Immunoprecipitations with anti-V5 antibody. The ubiquitin-positive smear band was increased in the mtRNP2 lane compared with that of the Wt.

To investigate the role of the RNP2 motif in the modification of TDP-43, we created two defective mutants, ΔRNP2 and mtRNP2 (Fig. 4A). While ΔRNP2 has no RNP2 motif, mtRNP2 contains the mutated RNP2 in which leucine residues were changed to aspartic acid, as previously reported [24]. The NSC34 cells overexpressing these mutants bear ubiquitin-positive inclusions in both the nucleus and cytosol (Fig. 4B, C). In immunocytochemical analyses, the inclusions of ΔRNP2 and mtRNP2 were distinct from RNA granules labeled with the anti-TIAR antibody (Fig. S4). The colocalization coefficient of mtRNP2 and ubiquitin was significantly higher than that of wild-type TDP-43 and ubiquitin (*p*<0.001; Fig. S5).

**Figure 5:**
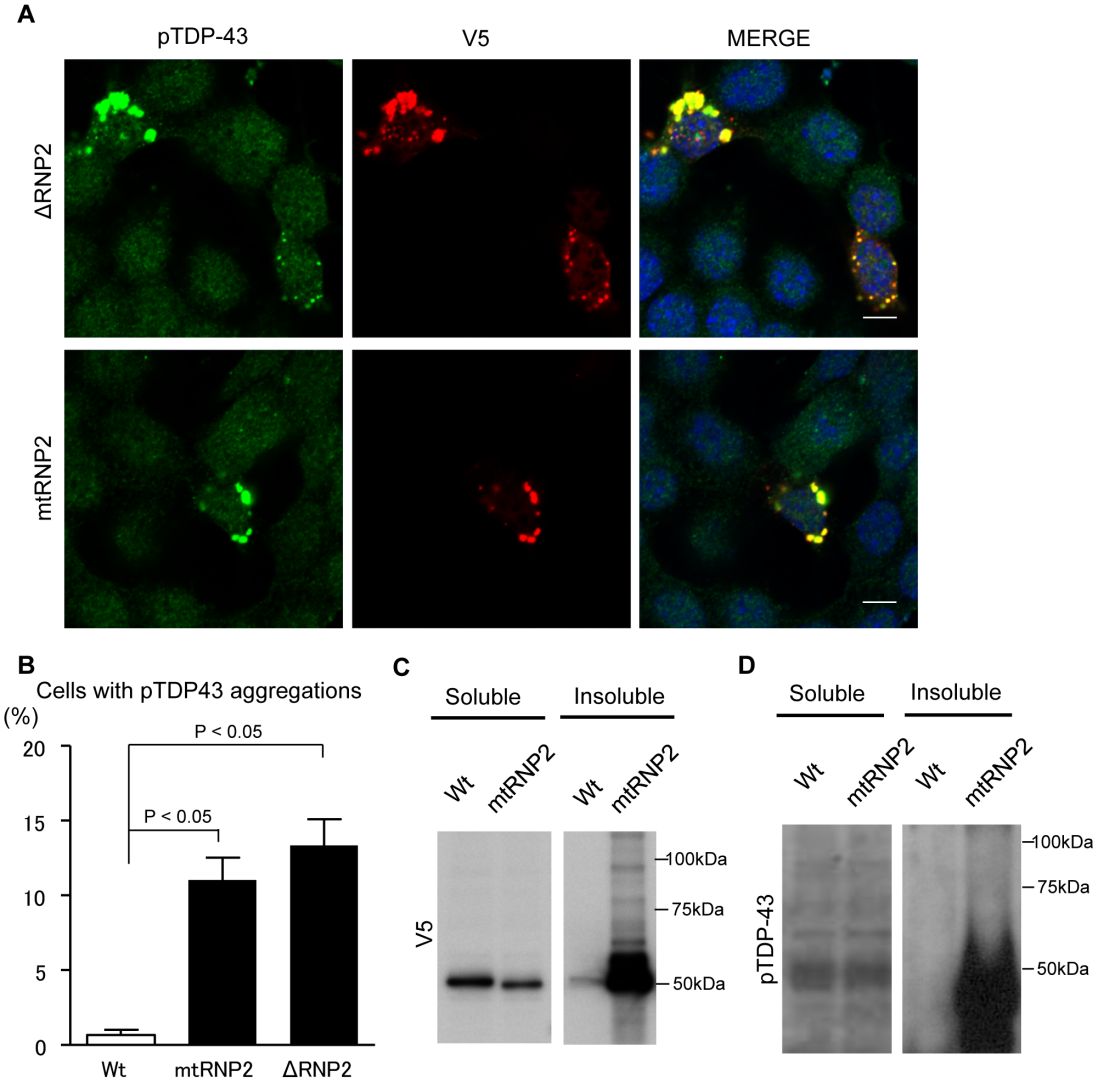
Phosphorylation and insolubilization of RNP2-disrupted TDP-43. (A) Immunocytochemistry of NSC34 cells expressing ΔRNP2 or mtRNP2. Cells were stained with anti-pTDP-43 antibody (green), anti-V5 antibody (red), and DAPI (blue). Scale bar = 5 µm. (B) Percentage of cells with pTDP-43-positive aggregates. Error bars indicate SEM (n = 3). The percentage of mtRNP2 and ΔRNP2-expressing cells containing pTDP-43-positive aggregates was significantly higher than that of Wt-expressing cells (*p*<0.01 and *p*<0.01, respectively). (C and D) Immunoblots of RIPA-soluble and -insoluble fractions from HEK293 cells expressing Wt or mtRNP2. The amount of insoluble mtRNP2 was higher than that of Wt (C). mtRNP2 in the RIPA-insoluble fraction was detected with anti-pTDP-43 antibody (D).

Immunoprecipitation analyses showed that mtRNP2 inclusions were strongly ubiquitinated in comparison with those of wild-type TDP-43 (Fig. 4D, E). Inclusions of ΔRNP2 and mtRNP2 were also immunoreactive to the anti-pTDP-43 antibody (Fig. 5A, B). The colocalization coefficient of mtRNP2 and pTDP-43 was significantly higher than that of wild-type TDP-43 and pTDP-43 (*p*<0.05; Fig. S6). The amount of mtRNP2 in the RIPA-insoluble fraction was higher than that of wild-type TDP-43 (Fig. 5C). Additionally, mtRNP2 was more phosphorylated than wild-type TDP-43 (Fig. 5D). These results further demonstrate the features of TDP32, supporting the view that RNP2 has a protective role for the pathological modification of TDP-43. Since V5 tag possibly has a certain effect on these modifications, we also assessed the biological properties of non-tagged mtRNP2 TDP-43. The results showed that mtRNP2 without a tag became also insoluble and hyperphosphorylated (Fig. S7A). In addition, we investigated TDP-43 with disrupted RNP1 motif (mtRNP1) in RRM1, which is also responsible for RNA binding of TDP-43. The mtRNP1 was insoluble and phosphorylated compared with wild-type TDP-43 (Fig. S7B).

### Biological Features of the 25 kDa CTF of TDP-43

**Figure 6:**
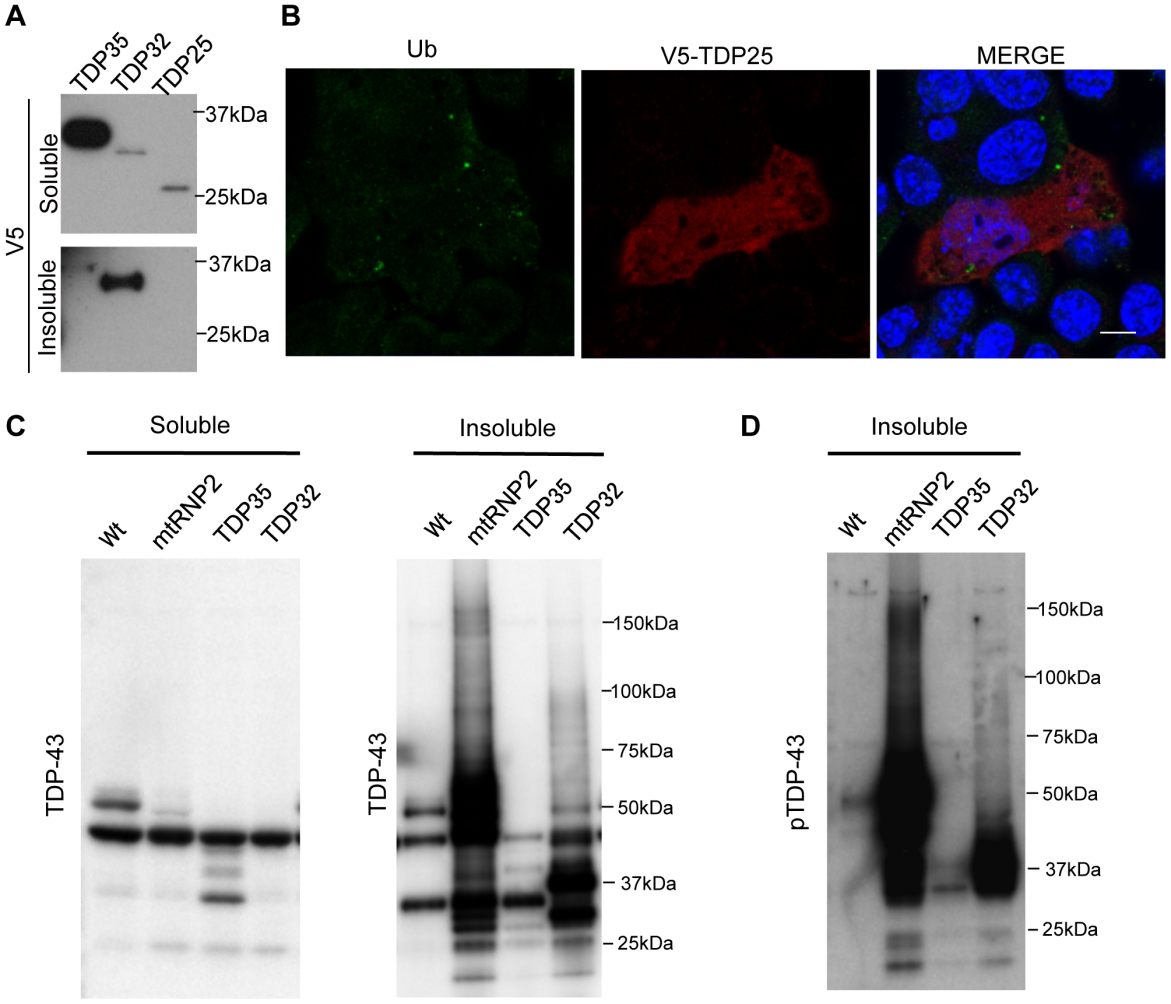
Biological features of the 25 kDa CTF of TDP-43. (A) Immunoblots of RIPA-soluble and -insoluble fractions from HEK293 cells expressing TDP35, TDP32, or TDP25. (B) Immunocytochemistry of cells expressing TDP25. Cells were stained with anti-ubiquitin (green), anti-V5 (red), and DAPI (blue). TDP25 was diffusely distributed and did not form aggregates. Scale bar = 5 µm. (C and D) Immunoblots of RIPA-soluble and -insoluble fractions from cells expressing Wt, mtRNP2, TDP35, or TDP32. Small fragments (∼26 kDa) of TDP-43 were detected in the lanes of mtRNP2 and TDP32 (C) and were immunoreactive to anti-pTDP-43 antibody (D).

Small CTFs of 18–26 kDa accumulate in the cytosol of affected neurons of TDP-43 proteinopathies [8], [25]. However, in the present cellular study, the 25 kDa CTF of TDP-43 (TDP25) was soluble (Fig. 6A) and scarcely formed ubiquitin-positive aggregates (Fig. 6B). These findings indicate that TDP25 is not insoluble when simply overexpressed in cultured cells. Given that TDP25 contains the complete RRM2 and C-terminal domains of TDP-43, our results suggest that these domains of TDP-43 do not by themselves play an essential role in aggregation. To confirm this hypothesis, we assessed the phenotype of C-terminal domain-lacking mtRNP2 mutants: mtRNP2 (1–273), which has RRM1 and RRM2; mtRNP2 (1–185), which contains RRM1 but not RRM2; and TDP-43 (1–105), which lacks both RRM1 and RRM2. The results showed that both mtRNP2 (1–273) and mtRNP2 (1–185) formed aggregates, whereas TDP-43 (1–105) lacking both RRMs diffusely located in the nucleus without aggregation (Fig. S8A, B). In addition, ΔRRM1 of TDP-43 did not apparently form aggregates (Fig. S8A, B). Although ΔRRM1 showed a punctate nuclear localization, it was not insoluble or phosphorylated in the immunoblots (Fig. S8C). Taken together, the disrupted RNP2 in conjunction with the remaining RRM1 is likely to be necessary for the aggregation of TDP-43.

Next we investigated whether the 18–26 kDa TDP-43 fragments are included in the aggregates of the CTFs we created. Following prolonged exposure of the immunoblots of TDP-43 mutants, we found that 25 kDa and shorter fragments were detected in the insoluble fraction of TDP32 and mtRNP2, but not TDP35, using pan-TDP-43 antibody (Fig. 6C). These fragments were also detected using the anti-pTDP-43 antibody that reacts with phosphorylated serines at the C-terminus of TDP-43 (Ser409/410) (Fig. 6D). Therefore, our findings indicate that the 25 kDa CTF of TDP-43 is included in the aggregation of TDP-43 mutants lacking the RNP2, though TDP25 does not by itself form aggregates. Since GFP-tagged TDP25 has been reported to form aggregates [26]–[28], we assessed the solubility of GFP-TDP-43 fragments. GFP-TDP35, TDP32, and TDP25 were all intensely insoluble (Fig. S9A). However, the features of non-tagged TDP-43 fragments were similar to those of V5-TDP-43 fragments: TDP32 was substantially insoluble, whereas TDP35 and TDP25 were less insoluble (Fig. S9B). These findings suggest that V5 tag appears to be suitable to assess the solubility of TDP-43 fragments.

### The 25 kDa CTFs Bind to TDP-43 Lacking the RNP2 Motif

**Figure 7:**
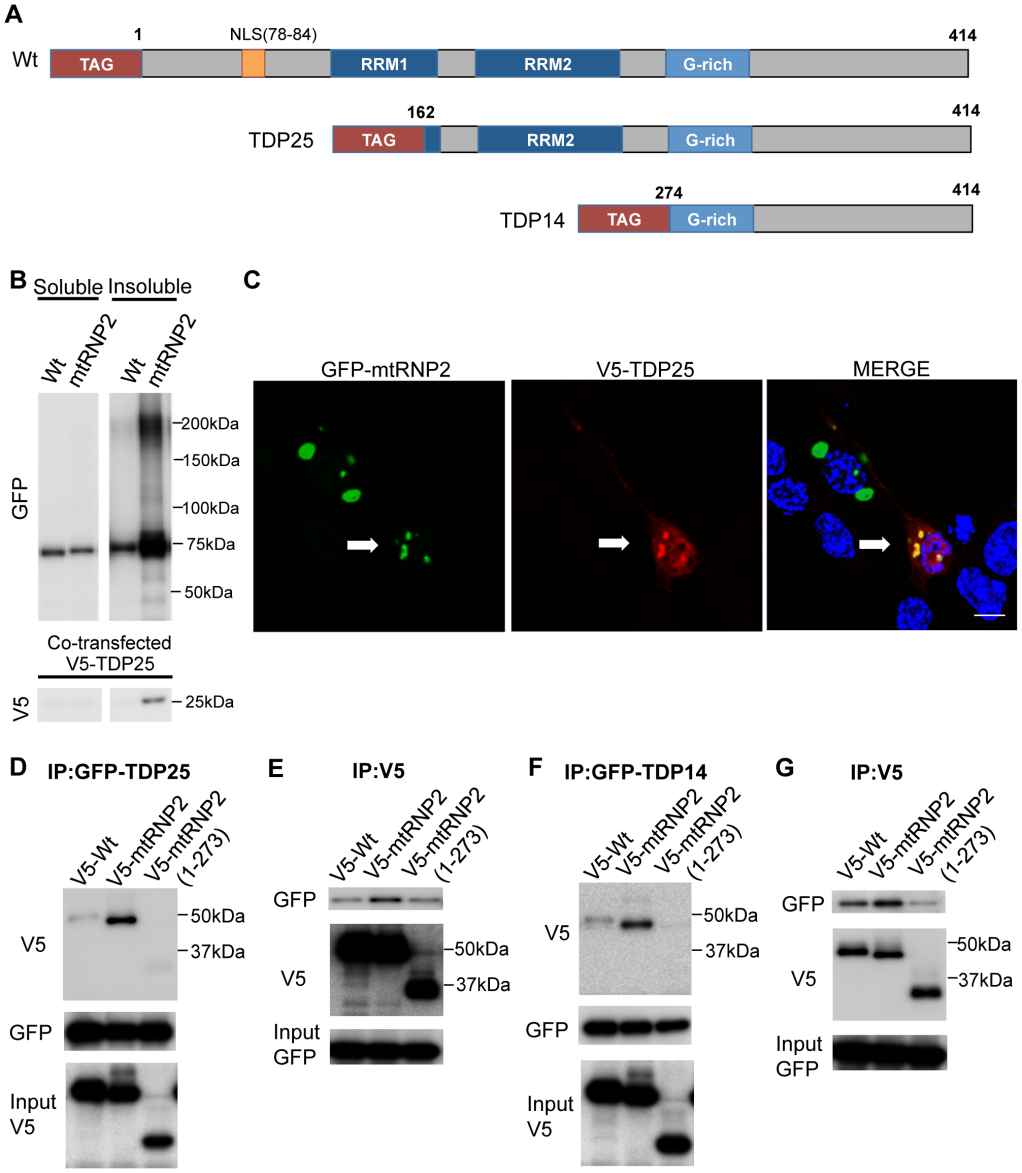
The 25 kDa CTF binds to TDP-43 lacking the RNP2 motif. (A) Structures of CTFs (wild-type, TDP25, and TDP14). (B) Immunoblots of RIPA-soluble and -insoluble fractions from HEK293 cells expressing GFP-Wt or mtRNP2. (C) Immunocytochemistry of NSC34 cells expressing GFP-mtRNP2 together with V5-TDP25. Cells were stained with anti-V5 (red) and DAPI (blue). Scale bar = 5 µm. (D and E) Immunoprecipitations with anti-GFP (D) or anti-V5 (E) antibody from cells expressing GFP-TDP25 and V5-mtRNP2. (F and G) Immunoprecipitations with anti-GFP (F) or anti-V5 (G) antibody from cells expressing GFP-TDP14 and V5-mtRNP2.

Since 25 kDa and shorter CTFs were detected in the insoluble fraction of mtRNP2, we assumed that mtRNP2 binds to, and thereby sequesters, the 25 kDa CTF. To test this hypothesis, experiments using the 25 kDa and 14 kDa CTFs of TDP-43 were performed (Fig. 7A). We co-transfected V5-tagged TDP25 and GFP-tagged TDP-43 in HEK293 cells and fractionated the whole cell lysates to obtain RIPA-soluble and -insoluble fractions. The amount of V5-TDP25 in the insoluble fraction was remarkably increased by GFP-mtRNP2, although we hardly detected the band of V5-TDP25 in the insoluble fraction of the cells expressing wild-type TDP-43 (Fig. 7B). Immunocytochemical confocal microscope analysis also demonstrated that V5-TDP25 was colocalized with the aggregates of GFP-mtRNP2 in the cytosol (Fig. 7C).

Next we performed immunoprecipitation to examine the binding of small CTFs to mtRNP2. The results showed that TDP25 binds to V5-mtRNP2, but not to the V5-mtRNP2 (1–273) that lacks the C-terminal domain of TDP-43 (Fig. 7D, E). Since TDP25 includes the RRM2 (aa 191–262), a shorter CTF, TDP14, was also used to determine whether the RRM2 is necessary for the binding to mtRNP2. TDP14, which spans amino acids 274–414, lacks RRM2, but contains the C-terminal region where most ALS-related mutations are located. The results showed that GFP-TDP14 efficiently binds to V5-mtRNP2 as well as GFP-TDP25 (Fig. 7F, G). In addition, we comfirmed that mtRNP2, as well as TDP35 and TDP32, showed no interaction with IgG/beads (Fig. S10). These results suggest that small CTFs, seen in the neurons of ALS and FTLD patients bind to the C-terminal domain of mtRNP2 and are sequestrated into the cytosolic aggregates of mtRNP2, and that the RRM2 is not required for this interaction. Although our results suggest that wild-type TDP-43 also interacts with the CTFs, this might result from the effect of the GFP tag given that GFP-TDP25 tends to be insoluble as shown in Fig. S7A.

### A Decrease in Cellular RNA Enhances the Insolubility of TDP-43

**Figure 8:**
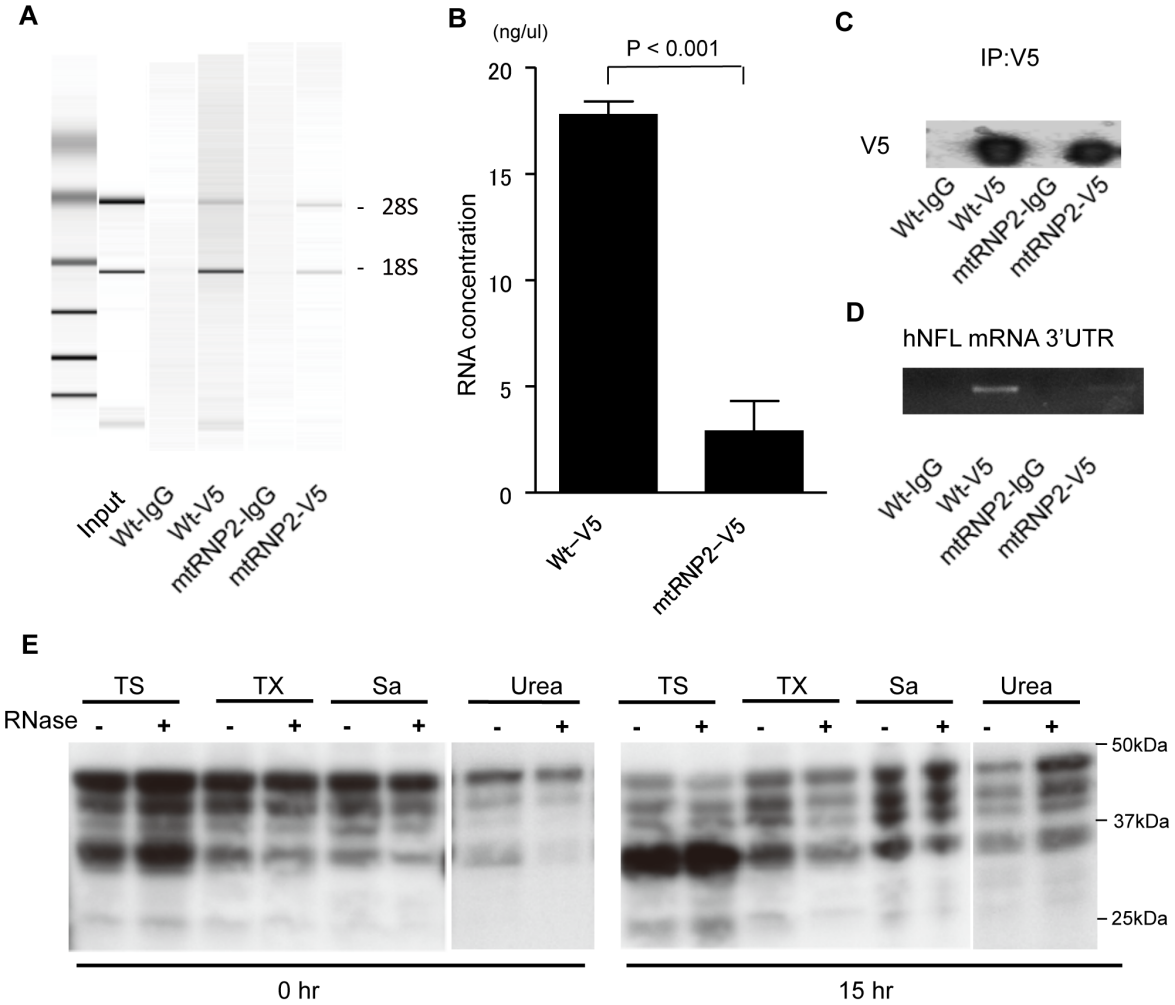
A decrease in cellular RNA increases the insolubility of TDP-43. (A) Electrophoresis of total RNA immunoprecipitated with wild-type TDP-43 or mtRNP2. (B) Results of RNA concentration analysis. The amount of RNA that binds to mtRNP2 was significantly decreased in comparison with wild-type TDP-43 (*p*<0.001). (C) Immunoblots of the immunoprecipitated protein with anti-V5 antibody or control IgG. (D) Reverse transcription-PCR (RT-PCR) of hNFL from immunoprecipitated RNA. The 3′UTR of hNFL was clearly detected by RT-PCR from RNA that binds to wild-type TDP-43. (E) Immunoblots of sequential extractions from cell lysates with or without RNase. The incubations with RNase induced insolubilization of TDP-43.

The RNP2 motif of RRM1 is important for its RNA binding ability [24]. To confirm that mtRNP2 lacks the ability to bind to RNA, we performed RNP immunoprecipitation. The results showed that the amount of RNA that binds to TDP-43 is decreased by the disruption of RNP2, although the efficiency of precipitation was similar between wild-type TDP-43 and mtRNP2 (Fig. 8A–C). We also tested whether mtRNP2 binds to the 3′UTR of the mRNA of human neurofilament light chain (NFL), a known target of TDP-43 [29], [30], using RNP immunoprecipitation followed by PCR. The results showed that wild-type TDP-43, but not mtRNP2, binds to the 3′UTR of NFL mRNA, confirming that mtRNP2 loses its ability to bind to the target RNA of TDP-43 (Fig. 8D).

Based on the observation that the disruption of the RNP2 motif increases the aggregation of TDP-43, we hypothesized that the decreased binding to RNA leads to the formation of insoluble aggregates of TDP-43. Therefore, we investigated the effects of RNase on the properties of the endogenous TDP-43. To increase the detection sensitivity of insolubilized TDP-43, we used a mild buffer and four-step fractionation. The results demonstrated that the amount of urea-insoluble endogenous TDP-43 was increased from that of the start sample (0 h) by a 16-h incubation with RNase (Fig. 8E).

## Discussion

The ubiquitin-positive, phosphorylated inclusion of TDP-43in neuronal cytoplasm is a pathological hallmark of TDP-43 proteinopathy. Since this inclusion contains 18–26 kDa CTFs of TDP-43 that do not have the RRM1 [8], [25], the phenotype of the TDP-43 CTFs have been intensively investigated. Previous studies showed that the 35 kDa TDP-43 CTF is sequestered into stress granules in cultured cells [31], [32], whereas the 25 kDa CTF phenotype findings have been controversial [25]–[28], [31], [33]–[35]: several reports did demonstrate that 25 kDa of TDP-43 forms cytosolic aggregates but most were tagged with fluorescent proteins. Therefore, a small tag, V5, was used to assess the cellular distribution of TDP-43 in our experiments.

We first focused on the physical features of two types of TDP-43 CTFs, TDP35 and TDP32. The cells expressing TDP32 only exhibited ubiquitin-positive and phosphorylated aggregations. In addition, the immunoblots also showed that TDP32, but not TDP35, was insoluble and phosphorylated, suggesting that the RNP2 motif in the RRM1 is responsible for the process of TDP-43 aggregation. Although the cells expressing TDP35 occasionally appeared to form cytoplasmic aggregates, those were the components of stress granules and were neither insoluble nor ubiquitinated. On the other hand, the cells expressing TDP25 did not form aggregates in our experimental conditions, although the TDP25 did not contain the RNP2 motif. This discrepancy might be explained by our observation that the disrupted RNP2 motif and the remaining RRM1, but not RRM2, are both required for the aggregations of TDP-43. In addition, the finding that ΔRRM1 TDP-43 does not form aggregates also supports this hypothesis.

Since the RNP2 motif in the RRM1 is responsible for the ability of TDP-43 to bind RNAs with specific sequences [24], a disruption of RNA binding could cause the aberrant aggregation of TDP-43. The cells with either ΔRNP2 or mtRNP2 TDP-43 formed aggregates and underwent both phosphorylation and ubiquitination. Disruption of RNP1, another RNA biding motif, also insolubilizes TDP-43. In addition, incubation with RNase caused insolubilization of endogenous TDP-43. These data further confirmed that RNA binding is important for the process of TDP-43 aggregation. Since the interaction of negatively charged RNA is responsible for the conformation of RNA-binding proteins, RNA may exert a chaperoning effect on its bound proteins [36]. Therefore, when the interaction of TDP-43 with RNA is disrupted, a consequent conformational change could cause TDP-43 to aggregate, and affect the function of NLS in this protein. Previous studies that demonstrated reduced levels of RNA in the motor neurons of ALS patients may support our hypothesis [37].

We have discussed the fact that the CTFs did not, except for TDP32, aggregate in normal conditions, whereas full-length TDP-43 could aggregate when RNA binding is disrupted. However, a question is raised as the major components of aggregated TDP-43 in TDP-43 proteinopathy patients are CTFs, such as TDP25. Although TDP25 did not by itself aggregate in our system, the cells expressing mtRNP2 or TDP32 contained ∼25 kDa phosphorylated TDP-43 in their insoluble fractions. In addition, in the cells co-expressing mtRNP2 and TDP25, TDP25 formed aggregates and colocalized with mtRNP2, suggesting that mtRNP2 sequesters TDP25 in the aggregations.

On the other hand, mtRNP2 (1–273), which lacks the C-terminal domain, did not have the ability to sequester TDP25. The C-terminal domain of TDP-43, in which most of the disease mutations are located, contains a glutamine/asparagine-rich (Q/N-rich) domain; also referred to as the prion-like domain, it is involved in the self-assembly of misfolded CTFs and the sequestration of TDP-43 into polyglutamine aggregates [38]–[40]. Therefore, it is possible that TDP-43 in which RNA binding is disrupted forms the initial aggregation core, and further sequestrates TDP-43 CTF into the aggregation through interactions with the C-terminal domain.

In summary, we demonstrated that the RNP2 motif in RRM1 plays a substantial role in pathological TDP-43 modifications and that disruption of RNA binding may underlie the process of TDP-43 aggregation.

## Supporting Information

Figure S1 **Immunocytochemistry of NSC34 cells expressing TDP35 or TDP32.** Cells were stained with anti-V5 (red) and anti-TIAR (green) antibodies. The aggregates with TDP35, but not TDP32, colocalized with TIAR. Scale bar = 5 µm.
(TIF)

Figure S2 **The colocalization coefficient of the ubiquitin and V5 signals.** Cells were stained with anti-V5 (green) and anti-ubiquitin (red) antibodies. Colocalization with ubiquitin was significantly higher in the cells expressing TDP32 than in those bearing TDP35 (*p*<0.001). Scale bar = 5 µm. Error bars indicate SEM (n = 3).
(TIF)

Figure S3 **The colocalization coefficient of the pTDP-43 and V5 signals.** Cells were stained with anti-V5 (green) and anti-pTDP-43 (red) antibodies. Colocalization with pTDP-43 was significantly higher in the cells bearing TDP32 than in those expressing TDP35 (*p*<0.001). Scale bar = 5 µm. Error bars indicate SEM (n = 3).
(TIF)

Figure S4 **Immunocytochemistry of NSC34 cells expressing ΔRNP2 or mtRNP2.** Cells were stained with anti-V5 (red) and anti-TIAR (green) antibodies. TIAR did not colocalize with the aggregates of ΔRNP2 or mtRNP2. Scale bar = 5 µm.
(TIF)

Figure S5 **The colocalization coefficient of the ubiquitin and V5 signals.** Cells were stained with anti-V5 (green) and anti-ubiquitin (red) antibodies. Colocalization with ubiquitin was significantly higher in the cells expressing mtRNP2 than in those bearing wild-type TDP-43 (*p*<0.001). Scale bar = 5 µm. Error bars indicate SEM (n = 3).
(TIF)

Figure S6 **The colocalization coefficient of the pTDP-43 and V5 signals.** Cells were stained with anti-V5 (green) and anti-pTDP-43 (red) antibodies. Colocalization with pTDP-43 was significantly higher in the cells expressing mtRNP2 than in those with wild-type of TDP-43 (*p*<0.05). Scale bar = 5 µm. Error bars indicate SEM (n = 3).
(TIF)

Figure S7 **Biological features of non-tagged mtRNP2 and V5-tagged mtRNP1.** (A) Immunoblots of RIPA-soluble and -insoluble fractions from HEK293 cells expressing non-tagged wild-type and mtRNP2 TDP-43. (B) Immunoblots of RIPA-soluble and -insoluble fractions from HEK293 cells expressing V5-tagged wild-type and mtRNP1 TDP-43.
(TIF)

Figure S8 **Intracellular localizations of N-terminal fragments of TDP-43 with mutated RNP2 and TDP-43 lacking RRM1.** (A) Structures of mtRNP2, mtRNP2 (1–273), mtRNP2 (1–185), TDP (1–105), and ΔRRM1 TDP-43. (B) Images of NSC34 cells expressing V5-mtRNP2 (1–273), mtRNP2 (1–185), TDP (1–105), and ΔRRM1 TDP-43. The cells bearing mtRNP2 (1–273) and mtRNP2 (1–185), but not mtRNP2 (1–105) or ΔRRM1, formed aggregates. Scale bar = 10 µm. (C) Immunoblots of RIPA-soluble and -insoluble fractions from HEK293 cells expressing wild-type and ΔRRM1.
(TIF)

Figure S9 **Effect of tag on TDP-43 insolubilization.** (A) Immunoblots of RIPA-soluble and -insoluble fractions from HEK293 cells expressing GFP-tagged wild-type and CTFs of TDP-43. (B) Immunoblots of RIPA-soluble and -insoluble fractions from HEK293 cells expressing non-tagged wild-type and CTFs of TDP-43.
(TIF)

Figure S10 **Lack of interaction between TDP-43 mutants and IgG/beads.** Immunoprecipitaions with mouse IgG from cells expressing wild-type and mutations of TDP-43.
(TIF)

Table S1 **Primers for TOPO cloning.**
(DOCX)

Table S2 **Primers for mutagenesis.**
(DOCX)
