# Supplementary figures and images for: RNP2 of RNA Recognition Motif 1 Plays a Central Role in the Aberrant Modification of TDP-43
Source: PLoS One. 2013 Jun 28;8(6):e66966. doi: 10.1371/journal.pone.0066966 (PMC3695991; doi:10.1371/journal.pone.0066966)

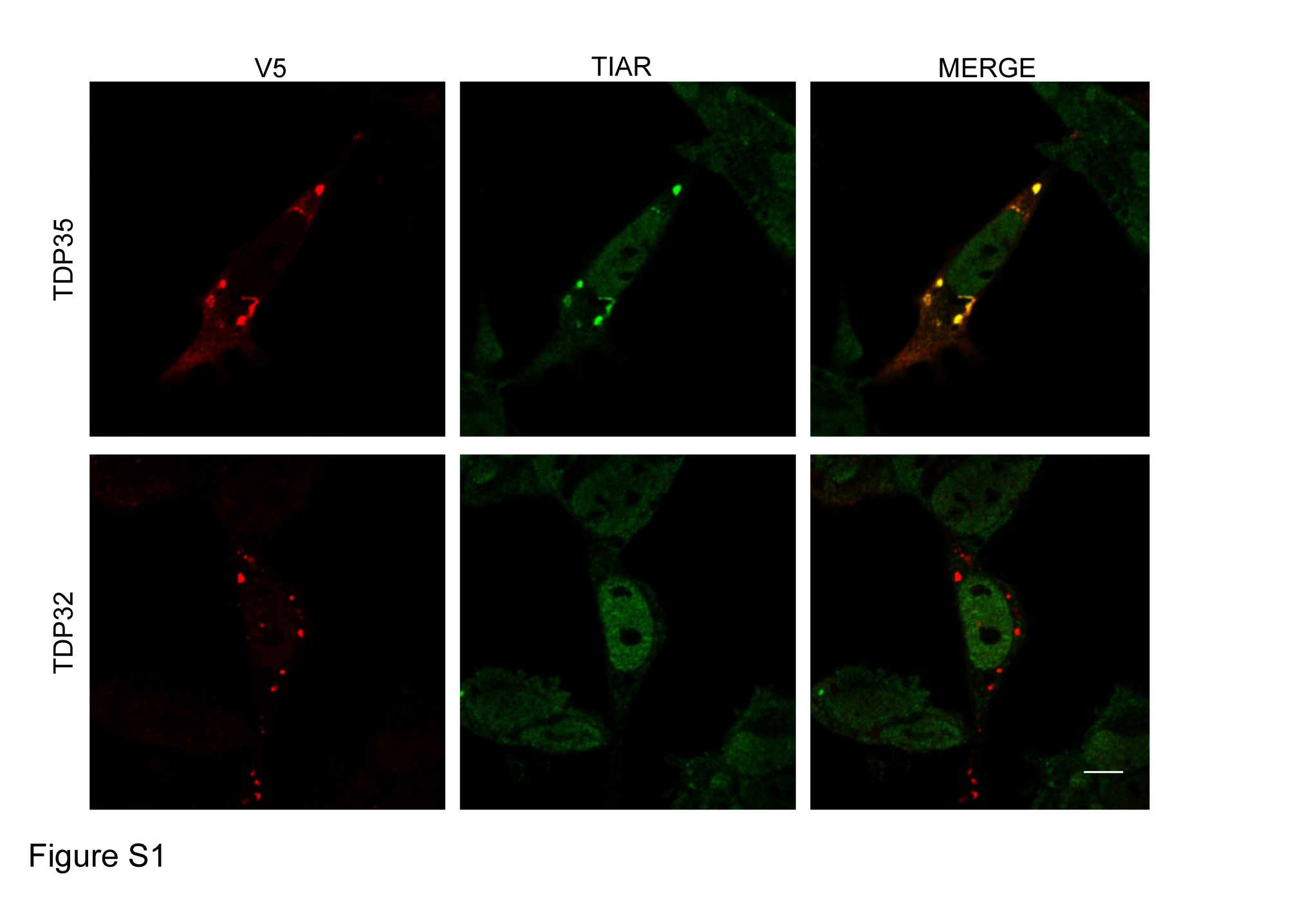

Supplement: Figure S1 — Immunocytochemistry of NSC34 cells expressing TDP35 or TDP32. Cells were stained with anti-V5 (red) and anti-TIAR (green) antibodies. The aggregates with TDP35, but not TDP32, colocalized with TIAR. Scale bar = 5 µm. (TIF) [file pone.0066966.s001.tif]

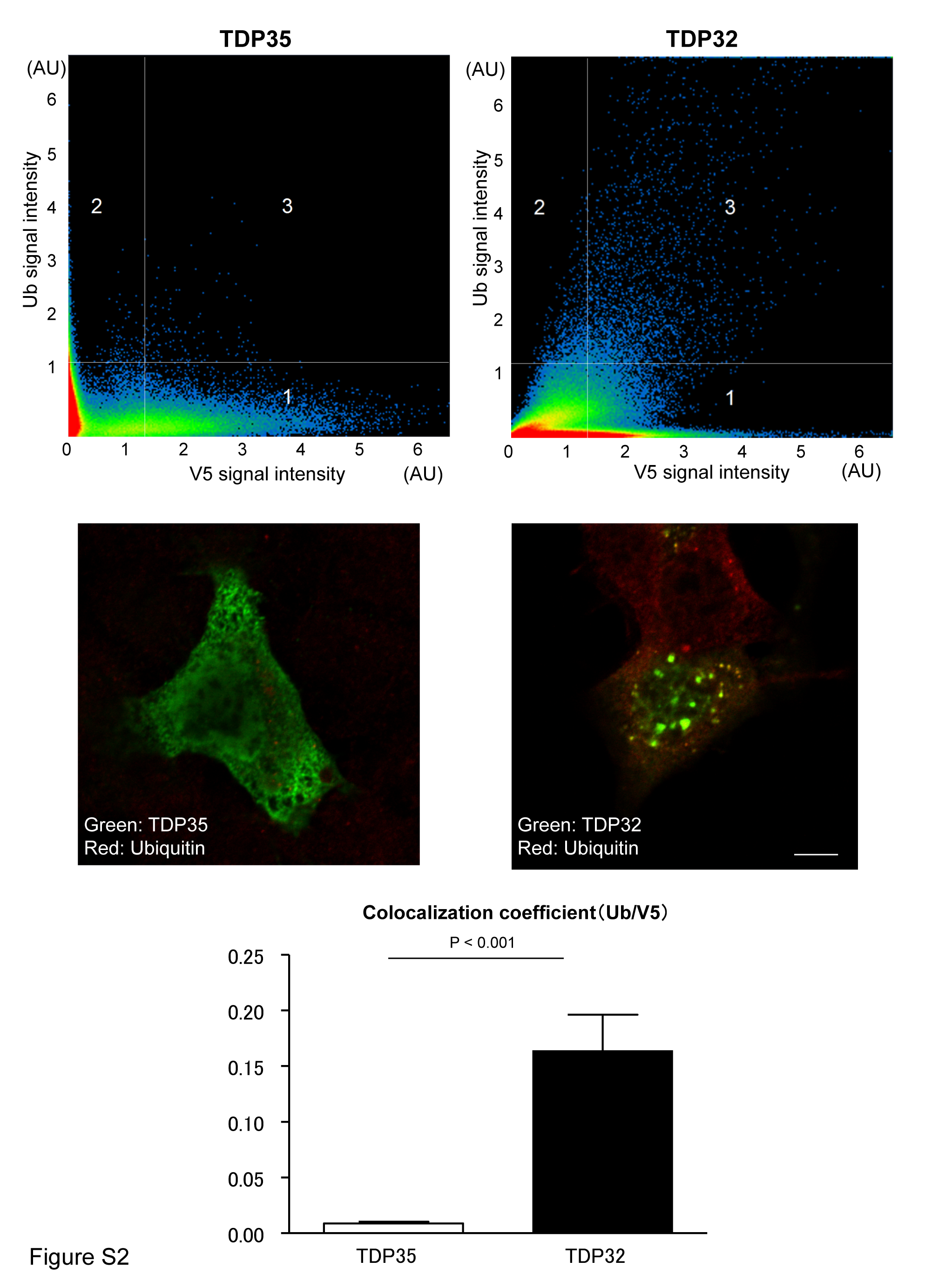

Supplement: Figure S2 — The colocalization coefficient of the ubiquitin and V5 signals. Cells were stained with anti-V5 (green) and anti-ubiquitin (red) antibodies. Colocalization with ubiquitin was significantly higher in the cells expressing TDP32 than in those bearing TDP35 (p<0.001). Scale bar = 5 µm. Error bars indicate SEM (n = 3). (TIF) [file pone.0066966.s002.tif]

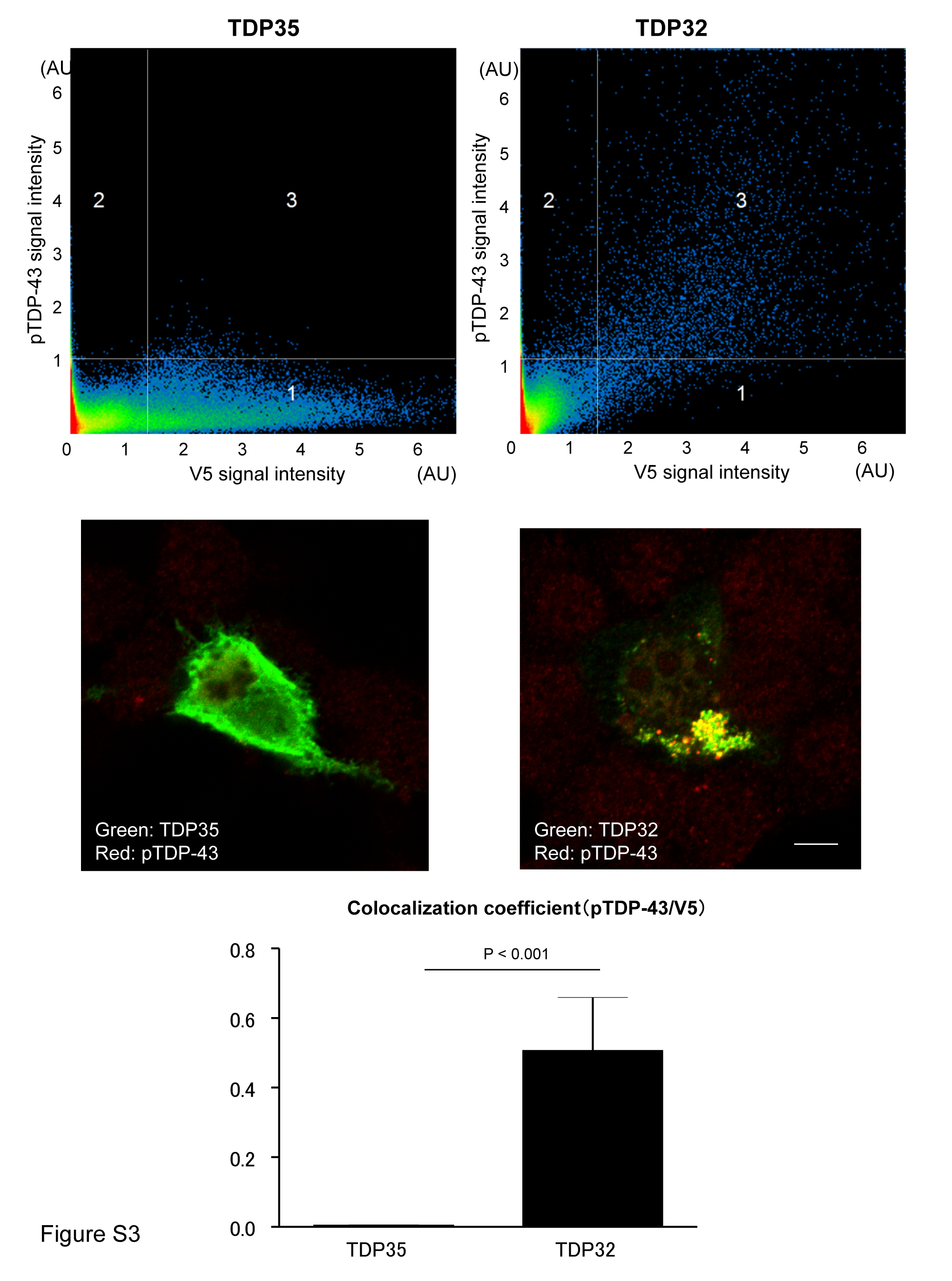

Supplement: Figure S3 — The colocalization coefficient of the pTDP-43 and V5 signals. Cells were stained with anti-V5 (green) and anti-pTDP-43 (red) antibodies. Colocalization with pTDP-43 was significantly higher in the cells bearing TDP32 than in those expressing TDP35 (p<0.001). Scale bar = 5 µm. Error bars indicate SEM (n = 3). (TIF) [file pone.0066966.s003.tif]

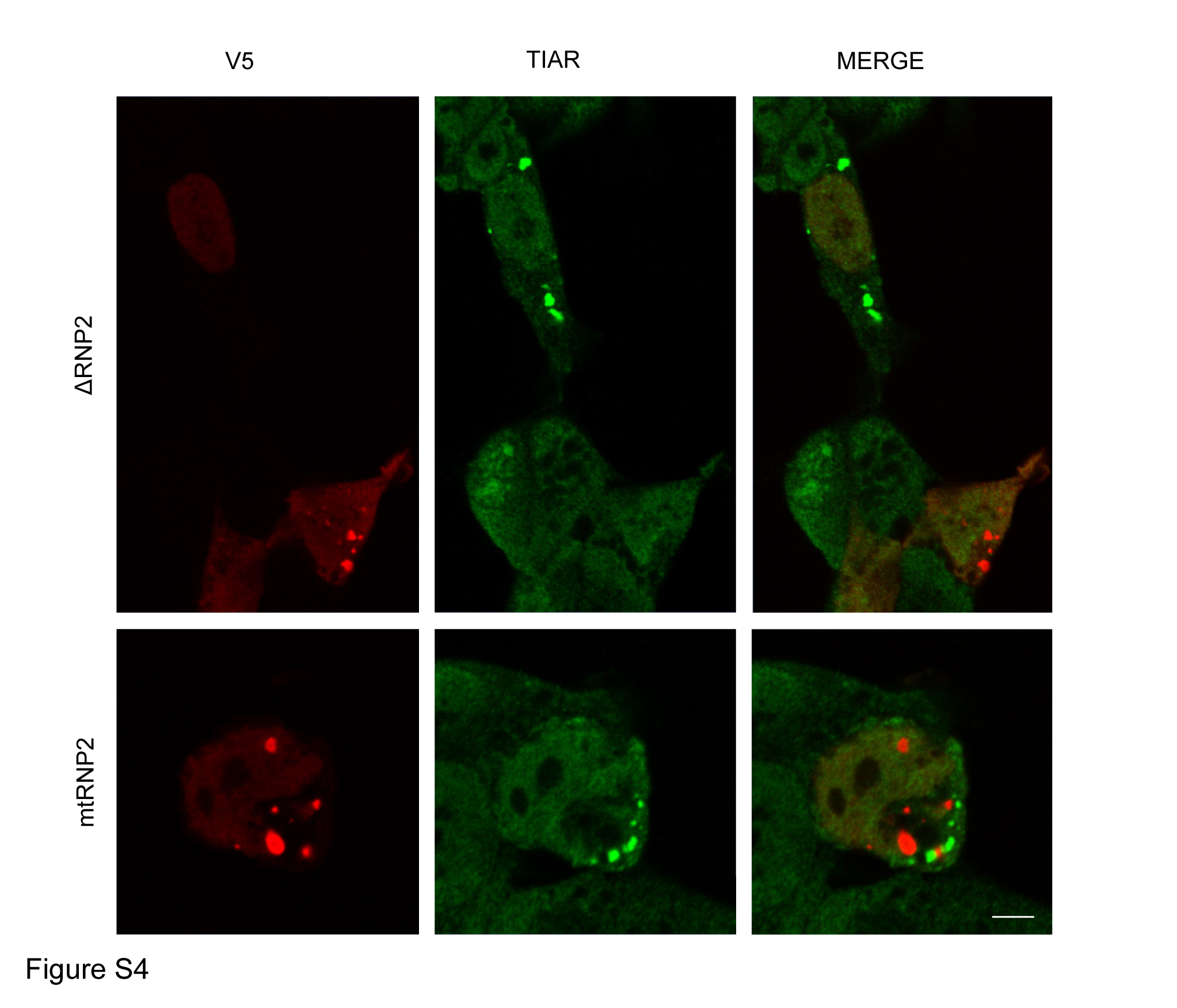

Supplement: Figure S4 — Immunocytochemistry of NSC34 cells expressing ΔRNP2 or mtRNP2. Cells were stained with anti-V5 (red) and anti-TIAR (green) antibodies. TIAR did not colocalize with the aggregates of ΔRNP2 or mtRNP2. Scale bar = 5 µm. (TIF) [file pone.0066966.s004.tif]

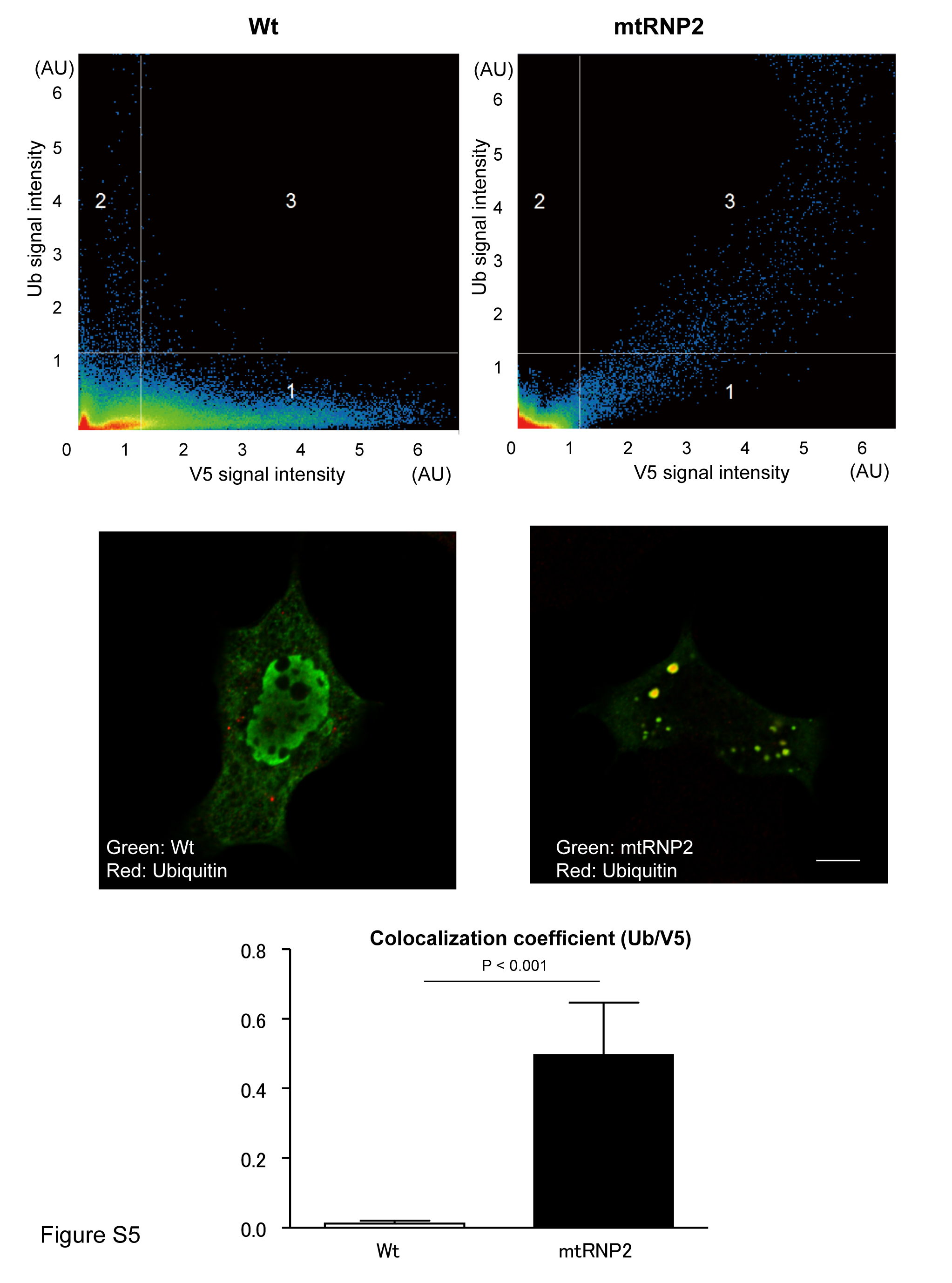

Supplement: Figure S5 — The colocalization coefficient of the ubiquitin and V5 signals. Cells were stained with anti-V5 (green) and anti-ubiquitin (red) antibodies. Colocalization with ubiquitin was significantly higher in the cells expressing mtRNP2 than in those bearing wild-type TDP-43 (p<0.001). Scale bar = 5 µm. Error bars indicate SEM (n = 3). (TIF) [file pone.0066966.s005.tif]

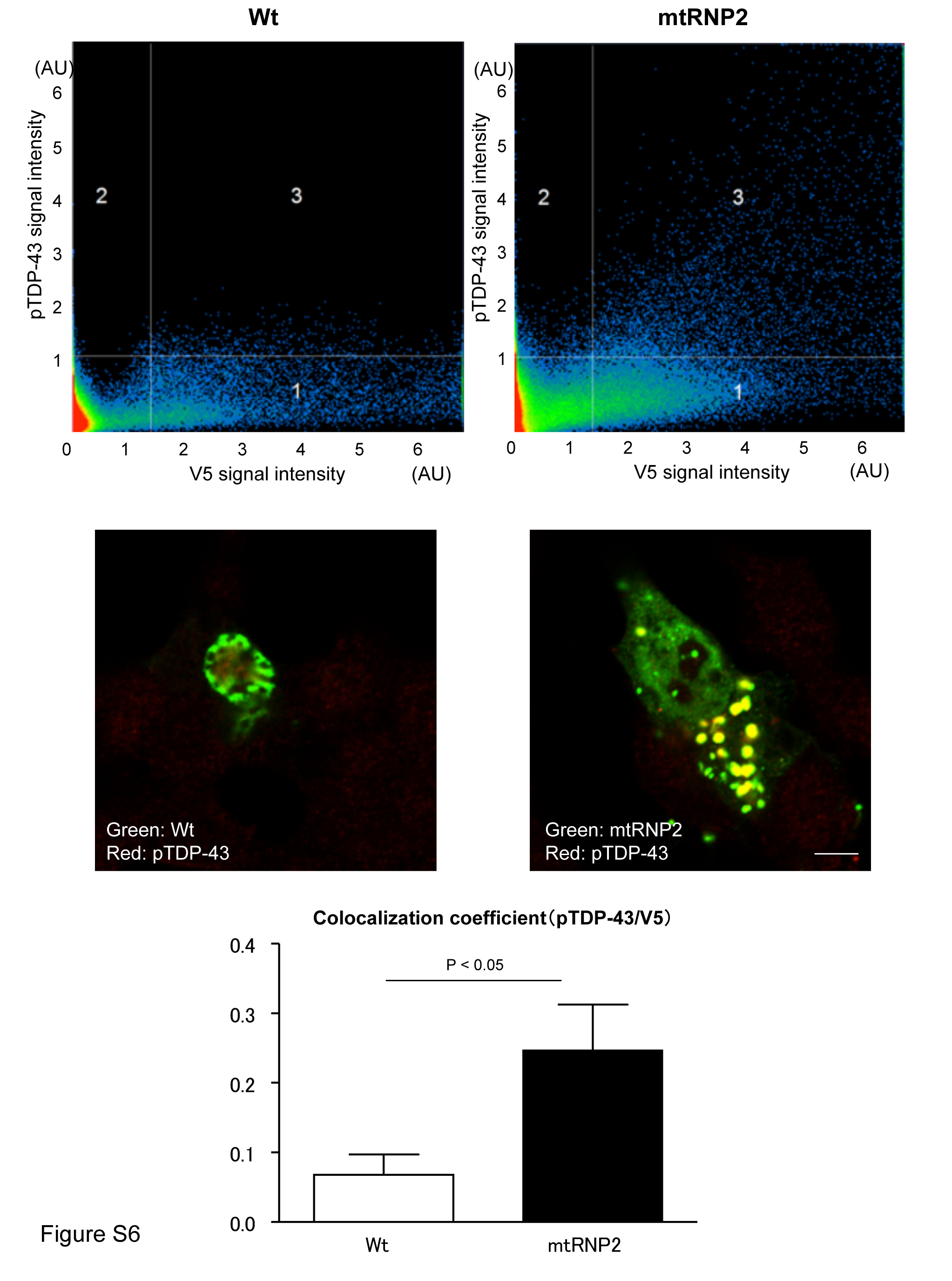

Supplement: Figure S6 — The colocalization coefficient of the pTDP-43 and V5 signals. Cells were stained with anti-V5 (green) and anti-pTDP-43 (red) antibodies. Colocalization with pTDP-43 was significantly higher in the cells expressing mtRNP2 than in those with wild-type of TDP-43 (p<0.05). Scale bar = 5 µm. Error bars indicate SEM (n = 3). (TIF) [file pone.0066966.s006.tif]

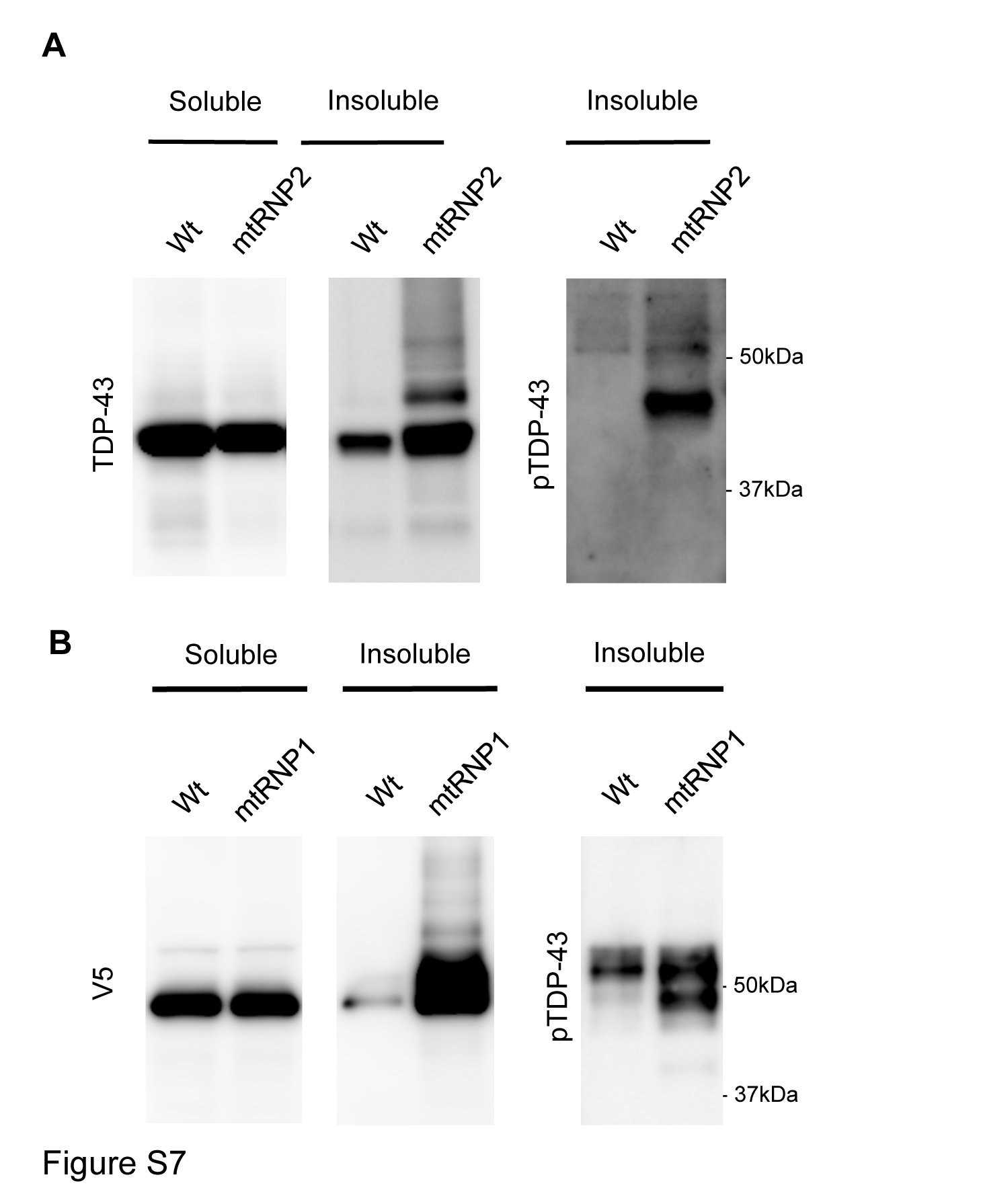

Supplement: Figure S7 — Biological features of non-tagged mtRNP2 and V5-tagged mtRNP1. (A) Immunoblots of RIPA-soluble and -insoluble fractions from HEK293 cells expressing non-tagged wild-type and mtRNP2 TDP-43. (B) Immunoblots of RIPA-soluble and -insoluble fractions from HEK293 cells expressing V5-tagged wild-type and mtRNP1 TDP-43. (TIF) [file pone.0066966.s007.tif]

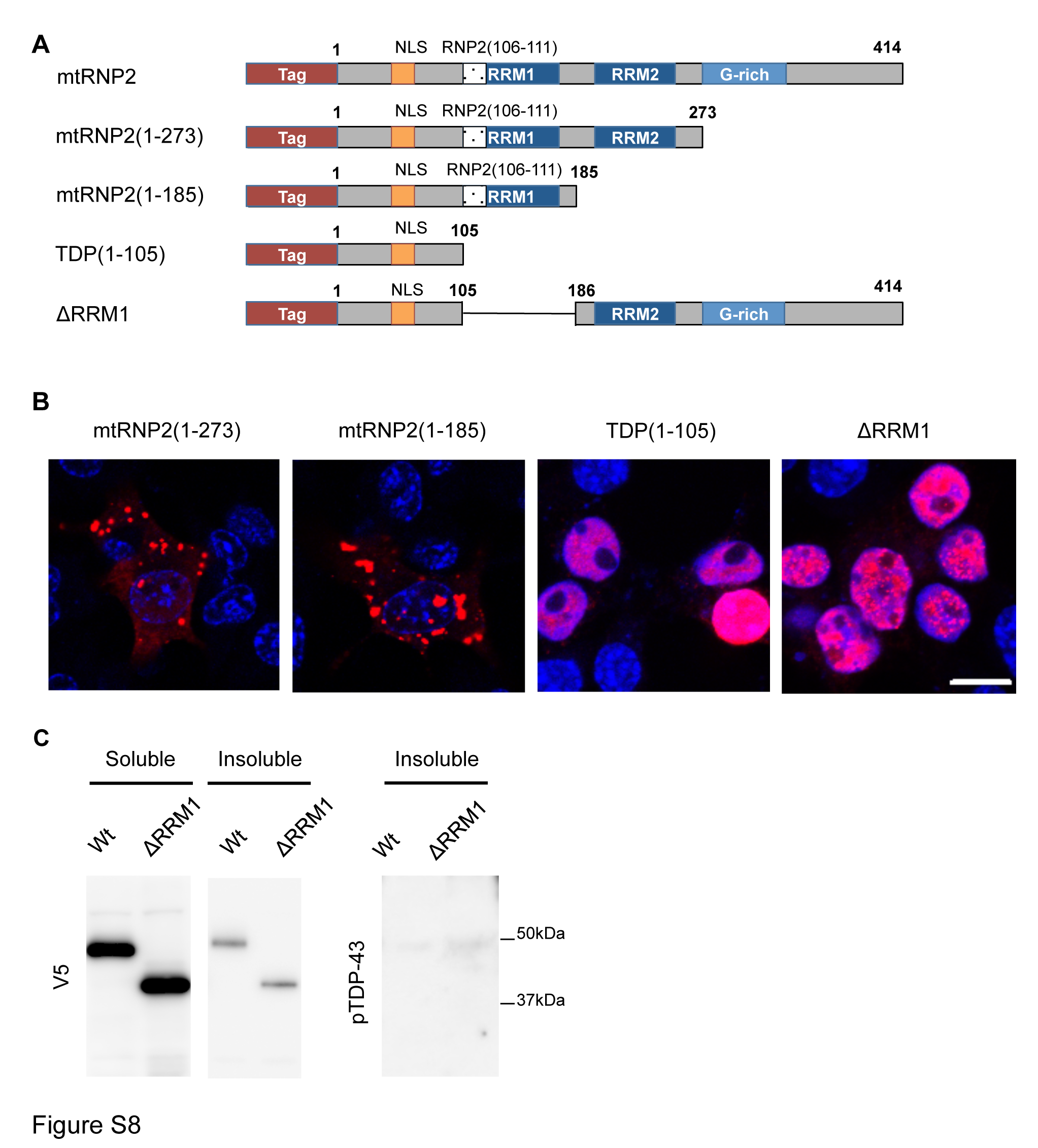

Supplement: Figure S8 — Intracellular localizations of N-terminal fragments of TDP-43 with mutated RNP2 and TDP-43 lacking RRM1. (A) Structures of mtRNP2, mtRNP2 (1–273), mtRNP2 (1–185), TDP (1–105), and ΔRRM1 TDP-43. (B) Images of NSC34 cells expressing V5-mtRNP2 (1–273), mtRNP2 (1–185), TDP (1–105), and ΔRRM1 TDP-43. The cells bearing mtRNP2 (1–273) and mtRNP2 (1–185), but not mtRNP2 (1–105) or ΔRRM1, formed aggregates. Scale bar = 10 µm. (C) Immunoblots of RIPA-soluble and -insoluble fractions from HEK293 cells expressing wild-type and ΔRRM1. (TIF) [file pone.0066966.s008.tif]

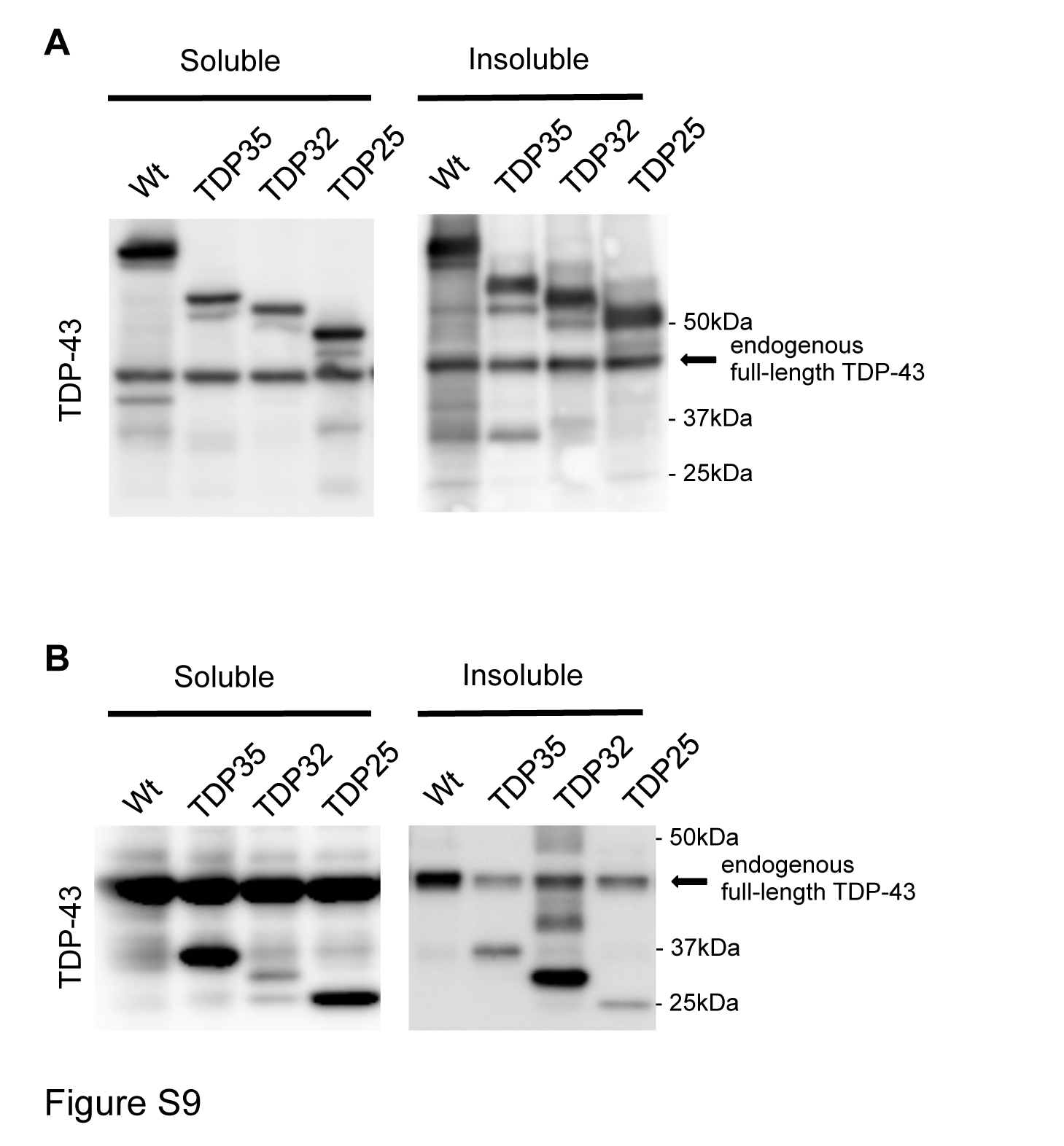

Supplement: Figure S9 — Effect of tag on TDP-43 insolubilization. (A) Immunoblots of RIPA-soluble and -insoluble fractions from HEK293 cells expressing GFP-tagged wild-type and CTFs of TDP-43. (B) Immunoblots of RIPA-soluble and -insoluble fractions from HEK293 cells expressing non-tagged wild-type and CTFs of TDP-43. (TIF) [file pone.0066966.s009.tif]

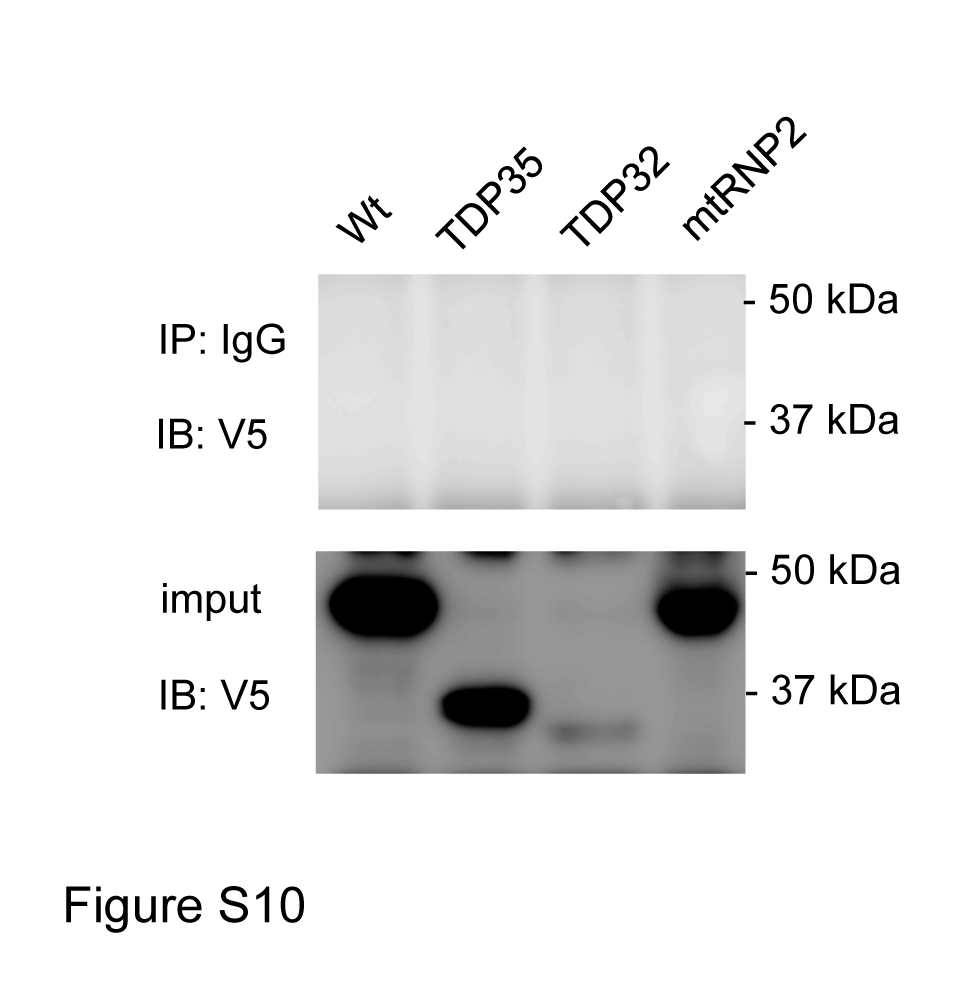

Supplement: Figure S10 — Lack of interaction between TDP-43 mutants and IgG/beads. Immunoprecipitaions with mouse IgG from cells expressing wild-type and mutations of TDP-43. (TIF) [file pone.0066966.s010.tif]
