# Supplementary material for: RNP2 of RNA Recognition Motif 1 Plays a Central Role in the Aberrant Modification of TDP-43
Source: PLoS One. 2013 Jun 28;8(6):e66966. doi: 10.1371/journal.pone.0066966 (PMC3695991; doi:10.1371/journal.pone.0066966)
Supplement: Table S1 — Primers for TOPO cloning. (DOCX) [file pone.0066966.s011.docx]

**Table S1 Primers for TOPO cloning.**

|  | Forward primer | Reverse primer |
| --- | --- | --- |
| TDP35 | CACCATGGATGAGACAGATGCTTCATCAGC | CTACATTCCCCAGCCAGAAGACTTAGAAT |
| TDP32 | CACC ATG CCATGGAAAACAACCGAACAGGACCTG | CTACATTCCCCAGCCAGAAGACTTAGAAT |
| TDP25 | CACCATGTCACAGCGACATATGATAGATGGACGATGGTGTGAC | CTACATTCCCCAGCCAGAAGACTTAGAAT |
| TDP14 | CACCGGAAGATTTGGTGGTAATCCAGGTGGC | CTACATTCCCCAGCCAGAAGACTTAGAAT |
| ΔRNP2  (1-273) | CAAAATGTCTGAATATATTCGGGTAAC | CTAACTTCTTTCTAACTGTCTATTGCT |
| mtRNP2 (1-273) | CAAAATGTCTGAATATATTCGGGTAAC | CTAACTTCTTTCTAACTGTCTATTGCT |
| mtRNP2 (1-185) | CAAAATGTCTGAATATATTCGGGTAAC | CTAATCTTGGCTTTGCTTAGAATTAGGAAG |
| TDP43 (1-105) | CAAAATGTCTGAATATATTCGGGTAAC | TCAATCGGATGTTTTCTGGACTGCTC |
