# Supplementary material for: RNP2 of RNA Recognition Motif 1 Plays a Central Role in the Aberrant Modification of TDP-43
Source: PLoS One. 2013 Jun 28;8(6):e66966. doi: 10.1371/journal.pone.0066966 (PMC3695991; doi:10.1371/journal.pone.0066966)
Supplement: Table S2 — Primers for mutagenesis. (DOCX) [file pone.0066966.s012.docx]

**Table S2 Primers for mutagenesis.**

|  | Forward primer | Reverse primer |
| --- | --- | --- |
| ΔRNP2 | CCATGGAAAACAACCGAACAGGACCTG | ATCGGATGTTTTCTGGACTGCTCTTTTCAC |
| mtRNP2 | ATTTGGGTGACCCATGGAAAACAACCGAACAGGACCTG | CTATATCATCGGATGTTTTCTGGACTGCTCTTTTCAC |
| dNLS | AATGGATGAGACAGATGCTTC | GCTGCTGCGTTATCTTTTGGATAGTTGACAACA |
| mtRNP1 | GCTTGGTTCGTTTTACGGAATATGAAACAC | CCAACCCCTTTGAATGACCAGTCTTAAGAT |
| dRRM1 | GAGCCTTTGAGAAGCAGAAAAGTGTTTGTGGGGCG | ATCGGATGTTTTCTGGACTGCTCTTTTCACTTTCAC |
